# Plant-Derived as Alternatives to Animal-Derived Bioactive Peptides: A Review of the Preparation, Bioactivities, Structure–Activity Relationships, and Applications in Chronic Diseases

**DOI:** 10.3390/nu16193277

**Published:** 2024-09-27

**Authors:** Li Sun, Jinze Liu, Zhongmei He, Rui Du

**Affiliations:** 1 College of Chinese Medicinal Materials, Jilin Agricultural University, Changchun 130118, China; 20221595@mails.jlau.edu.cn (L.S.); liujinze0602@mails.jlau.edu.cn (J.L.); 2 Jilin Provincial Engineering Research Center for Efficient Breeding and Product Development of Sika Deer, Changchun 130118, China

**Keywords:** plant-derived bioactive peptides, enzymolysis, bioactivities, structure–activity relationship, chronic diseases

## Abstract

**Background/Objectives:** At present, a large number of bioactive peptides have been found from plant sources with potential applications for the prevention of chronic diseases. By promoting plant-derived bioactive peptides (PDBPs), we can reduce dependence on animals, reduce greenhouse gas emissions, and protect the ecological environment. **Methods:** In this review, we summarize recent advances in sustainably sourced PDBPs in terms of preparation methods, biological activity, structure–activity relationships, and their use in chronic diseases. **Results:** Firstly, the current preparation methods of PDBPs were summarized, and the advantages and disadvantages of enzymatic method and microbial fermentation method were introduced. Secondly, the biological activities of PDBPs that have been explored are summarized, including antioxidant, antibacterial, anticancer and antihypertensive activities. Finally, based on the biological activity, the structure–activity relationship of PDBPs and its application in chronic diseases were discussed. All these provide the foundation for the development of PDBPs. However, the study of PDBPs still has some limitations. **Conclusions:** Overall, PDBPs is a good candidate for the prevention and treatment of chronic diseases in humans. This work provides important information for exploring the source of PDBPs, optimizing its biological activity, and accurately designing functional foods or drugs.

## 1. Introduction

With increased awareness of environmental pollution, animal cruelty, and the negative health effects of animal-based foods, consumers are turning to plant-based vegan alternatives [1]. The EAT-Lancet Commission report also points to a shift towards more plant-based diets to meet the UN Sustainable Development Goals and the Paris Agreement, as well as using the Earth Healthy Diet as a reference for healthy and sustainable diets for a growing population [2]. In recent decades, plant-derived bioactive peptides (PDBPs) have attracted much attention due to their low cost and environmental sustainability. The use of plants as ingredients in food and other industries is widely accepted worldwide, so there is no religious or sociocultural prejudice against their use [3]. In addition, sources of PDBPs include legumes, grains, nuts, fruits, vegetables, and so on [4,5]. Compared with animal sources, PDBPs have abundant sources, simple preparation and low cost, and have a good development trend [6,7].

It has been reported that PDBPs can obtain small bioactive peptides by enzymatic hydrolysis, microbial fermentation, chemical synthesis and other methods under specific conditions [8,9]. Among them, enzymatic hydrolysis and microbial fermentation are the most favorable biotechnological methods for releasing bioactive peptides [10]. PDBPs have specific biological functions that original proteins and amino acids do not possess. Their various biological activities, such as hypotensive, antioxidant, antibacterial, hypoglycemic, and anticancer properties, are constantly being explored [11]. The biological activity of PDBPs is related to their specific amino acid composition, sequence, quantity, position in the carbon chain and the spatial structure of the peptide chain [12]. Relevant studies have pointed out that PDBPs have the advantages of a low molecular weight and easy digestion and absorption in the intestine [13,14]. These characteristics determine their function to a certain extent, and, thus, have more favorable treatment effects for chronic diseases. For example, peptides extracted from rice bran inhibit the growth of bowel cancer cells (Caco-2), breast cancer cells (MCF-7), and liver cancer cells (HepG-2) [15]. In addition, peptides derived from grains, such as oats and barley, have a strong inhibitory effect on ACE [16]. Studies have found that PDBPs can be used to prevent and treat cancer, high blood pressure, and other chronic diseases. They have high edible and medicinal value and can meet the various needs of human health [17].

**Figure 1:**
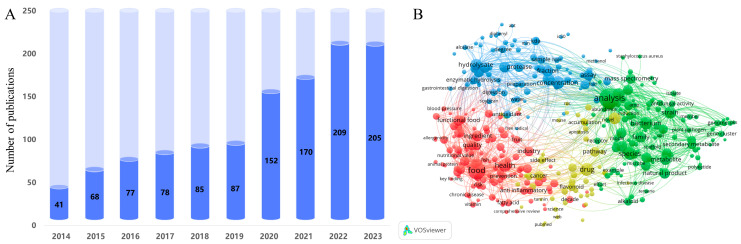
(**A**) Number of PDBPs-related publications in the Web of Science core collection in the past decade. (**B**) Keyword co-occurrence map based on bibliographic data created through VOSviewer 1.6.20 software (PDBPs, 2014–2023).

Notably, over the past decade, mentions of PDBPs have increased from 41 in 2014 to 205 in 2024 (Figure 1A). Figure 1B shows the co-occurrence of keywords associated with PDBPs. Among them, there are many keywords related to human chronic diseases, such as inflammation, blood pressure, cancer, and chronic diseases. It can be seen that researchers are increasingly exploring PDBPs, and the unique structural characteristics and functional mechanisms of PDBPs are increasingly attracting attention. Therefore, based on the latest research progress of PDBPs in recent years, this review focuses on the biological activity, structure–activity relationship, and application of PDBPs in chronic diseases. The preparation methods and limitations of PDBPs are also briefly introduced. It is expected to provide reference for further research and application development of PDBPs.

## 2. Methods

This narrative review searched the PubMed, Web of Science, Google Scholar, SpringerLink, and Science Direct databases using keywords and related terms. It used certain keywords, i.e., plants and bioactive peptides, and combined such terms with the following keywords: enzymolysis, fermentation, bioactivity, structure–activity relationship, and chronic diseases. The final search was conducted in June 2024. The literature search language was English, and references were selected according to their relevance. In this review, we first read the title of the high-value references in the literature, then searched by the title, and finally read the whole article. References that were not relevant to the review and replicated studies were excluded, and abstracts of the remaining articles were reviewed to ensure that they met the review’s inclusion criteria. Because this article is a narrative review, it is not necessary to document the literature searches on specific platforms [18].

## 3. Preparation of PDBPs

According to the different internal and external factors, such as environment, cost, and sample composition, different preparation methods are selected to obtain better PDBPs. At present, the common preparation methods of PDBPs include enzymatic hydrolysis and microbial fermentation. Therefore, we briefly introduce the advantages and disadvantages of the following two preparation methods (Table 1).

### 3.1. Enzymatic Hydrolysis

Enzymatic hydrolysis is one of the main methods of protein hydrolysis in the food industry. Its mild reaction conditions, high selectivity, and high safety have been proven to be an economical and effective strategy [19]. Its main method is to hydrolyze the amide bond sites of proteins by specific enzymes, so as to obtain different types of peptides, which is the most commonly used method for preparing plant bioactive peptides. Because the specificity of different enzymes is different, some low molecular peptides with biological activity can be obtained by using one or more proteolytic enzymes [20].

#### 3.1.1. Monoenzymatic Hydrolysis

In the study of monoenzyme hydrolysis, alcalase can specifically break peptide bonds. Therefore, compared with other enzymes, alcalase is often used for proteolysis from various plant sources. An et al. [21] proved that the optimal conditions for the preparation of wheat flour polypeptide by enzymolysis (alcalase) were as follows: the ratio of solid to liquid was 1:7, the amount of enzyme was 1.2%, the reaction time was 2 h, and the yield and purity of the peptide could reach 83% and 72%, respectively. In addition, Xiang and colleagues [22] obtained the best conditions for preparing seabuckthorn seed meal protein hydrolysate using alcalase: an initial pH = 10, a temperature of 50 °C, an enzyme dosage of 0.25 g/100 g protein, and a hydrolysis time of 8 h. Cottonseed protein hydrolysate obtained by alcalase showed antibacterial activity against four tested strains (*Staphylococcus aureus*, *Streptococcus*, *Escherichia coli*, and *Salmonella*) [23]. Golden melon seeds were hydrolyzed with alkaline protease to obtain peptide. Frac1 (molecular weight > 5 kDa), Frac2 (molecular weight 3~5 kDa), and Frac3 (molecular weight < 3 kDa) were obtained by ultrafiltration. Frac3 had the highest antioxidant capacity [24].

Other proteases are also used in the experiments of single enzymatic hydrolysis of proteins. Some scholars have studied and compared the enzymatic hydrolysis of a variety of different enzymes on the same plant. Tian et al. [25] used microwave-assisted enzymatic hydrolysis of wheat germ albumin to study the effects of alkaline protease, neutral protease, papain, and compound protease on its physical and chemical properties. The results showed that papain had the best proteolytic activity. The solubility increased and the apparent viscosity and foam stability decreased. A recent study found that hydrolyzed protein isolates from pigeon peas, lentils, and chickpeas could be extracted using alcalase and bromelain, and the study found that bromelain enhanced the water absorption and oil binding capacity of the three proteins. The potential of enzymatic hydrolysis in the production of functional components with antioxidant and anti-inflammatory properties in legume proteins was concluded [26]. In another study, Ren et al. analyzed the effects of five food-grade proteases, namely alkaline protease, neutral protease, trypsin, pepsin, and trypsin, on the antioxidant activity of rice bran protein hydrolysate. The results showed that trypsin hydrolysate had high iron chelation activity, DPPH and hydroxyl radical scavenging activity, and could improve the oxidative damage of Caco-2 cells induced by hydroxyl radicals [27]. In addition, in 2022, some scholars studied the five kinds of enzyme hydrolysis of mung bean protein enzymatic hydrolysis ability, including alcalase, neutrase, flavorzyme, papain, and protamex, and proved the highest alcalase hydrolysis degree. The physicochemical properties of five enzymatic hydrolysis products were evaluated by SDS-PAGE, particle size distribution analysis, FTIR, and ultraviolet visible and fluorescence spectrophotometry. It was also found that the products of protamex and papain had a higher foaming ability, emulsifying activity index, and emulsifying stability index [28]. The hydrolysates and fractions of *Ulva* sp. produced with food-grade papain have low renin and high ACE-1 inhibitory activities in vitro [29]. Similarly, Lu et al. [30] also used a variety of enzymatic hydrolysis comparison methods to explore the zinc-binding ability and in vitro gastrointestinal stability of Cucurbita pepo L after hydrolysis by different enzymes. The peptides hydrolyzed by papain had the largest average molecular weight, the smallest particle size, the highest hydrophobicity, and the largest zinc binding ability, and showed better stability.

#### 3.1.2. Complex Enzymatic Hydrolysis

Complex enzymatic hydrolysis is based on the action of a single enzyme and the combination of one or more proteases, according to the simultaneous hydrolysis of proteins or step-by-step hydrolysis of complex enzymatic hydrolysis. In 2024, an article was published on the dual enzyme hydrolysis of walnut pomace, which used aminopeptidase combined with alcalase hydrolysis to study the effect of different digestion methods on the flavor and antioxidant activity of walnut pomace protein products [31]. Chia seed by-products were hydrolyzed using alcalase and flavorzyme, and then hydrolyzed with alcalase and flavorzyme, respectively. It was found that chia seed protein obtained by using two enzymes (alcalase and flavorzyme) had the highest hydrolysis degree, and potential antioxidant, antihypertensive, and antithrombotic peptides could be obtained [32]. Other studies have shown that the combined enzymolysis of *Moringa oleifera* leaf protein (alcalase, neutrase, and flavorzyme) has stronger ACE inhibitory activity and DH than a single enzymolysis [33]. The optimal semi-solid enzymatic hydrolysis (SEH) parameters obtained for the preparation of adzuki bean peptides by SEH incorporating antioxidant activity, ACE inhibitory activity, and enzymatic efficiency included a ratio of alkaline protease–neutral protease = 2:1 (*w*/*w*) and enzyme addition of 3.75% (*w*/*w*) [34]. In addition, it was found that three soybean peptide groups prepared by different methods (alcalase + flavorzyme: hydrophobic amino acid-rich peptides; papain and flavorzyme: basic amino acid-rich peptides; and bromelain and flavorzyme: balanced amino acid compositions) had different sequence characteristics for their endonuclidenase cleavage sites [35]. Bioactive peptides with increased ACE inhibition and antioxidant activity have been produced by single and sequential hydrolysis of green lentil (*Lens culinaris*) seed. The results show that using a combination of enzymes in continuous hydrolysis can produce bioactive peptides with improved ACE inhibition and antioxidant activity, and with better antihypertensive and antioxidant activity [36]. It has been reported that using three different proteases (alkaline protease, papain, and trypsin), an encrypted and highly potent ACE-inhibitory peptide can be released from defatted moth bean powder from moth bean seeds [37].

#### 3.1.3. Microbial Fermentation

Microbial fermentation is a relatively old food biological technology [38]. It is not only an inexpensive way to produce bioactive peptides from protein substrates, but also to remove hyper-allergenic or anti-nutritional factors that may be present [39,40]. In the food industry, in order to obtain PDBPs with good physical and chemical properties, fermentation is also used to obtain stable and high-quality products [41]. The bioactive peptides obtained by the fermentation of red lentil protein isolate with various lactic acid bacteria and yeast strains have anti-free radical, angiotensin-converting enzyme inhibiting, and antifungal activities [42]. Serena et al. [43] used strains of lactobacillus to ferment a new pistachio beverage. According to the BIOPEP database, the resulting peptides were found to have potential biological activity, mainly related to antioxidant properties and ACE and DPP-IV inhibition. In addition, an in vitro experimental study found that the mixed fermentation of lactic acid bacteria and Aspergillus oryzae was used to prepare soybean meal. The optimal fermentation conditions were determined by response surface analysis. DPPH and ABTS free radical assays were used to determine the higher antioxidant activity of soybean peptides. At the same time, it was also found that it can effectively inhibit the hemolysis of red blood cells induced by AAPH, prevent the production of intracellular ROS, balance the antioxidant enzymes (SOD, CAT, and GSH-Px) system, and reduce the expression of MDA content [44]. *Brassica napus* is one of the top ten oil crops in the world, and after being pressed to make oil, the remainder is called the rapeseed meal. More recently, rapeseed meal has been fermented by a mixture of strains (*Bacillus subtilis, Pediococcus acidilactici*, and *Candida tropicalis*). Compared with the unfermented samples, the microbial diversity decreased significantly, indicating that mixed bacteria fermentation could inhibit the growth of mixed bacteria and increase the polypeptide content [45].

### 3.2. Separation and Purification

Plant protein hydrolysates and peptides can be separated by molecular weight, and low-molecular-weight protein hydrolysates often have better biological activity and can be used for further study. The protein hydrolysis product obtained after purification is purer, removing impurities and unhydrolyzed large proteins from the mixture [46]. The separation and purification of peptide mixtures have been well validated under laboratory conditions. However, the time required to go from enzymatic hydrolysis to separation and purification is long and the equipment is expensive. More exploration and process optimization are needed to improve the process. At present, the separation and purification methods of peptides used in the market include gel filtration chromatography, ion exchange chromatography, reversed-phase high-performance liquid chromatography (RP-HPLC), capillary electrophoresis (CE), ultrafiltration (UF), nano-liquid chromatography–mass spectrometry (nano LC-MS/MS) and other methods [47]. The separation and purification of peptides is usually first performed by ultrafiltration [48]. The technical principle is to remove macromolecular impurities by membrane separation technology, so as to obtain peptides of different molecular weights with different biological activities. However, due to the use of ultrafiltration technology, further separation and purification of the enzyme products are required. Therefore, it is difficult to obtain high purity peptides for further purification by chromatography [49]. For example, Dou et al. [50] isolated and purified peptides from Idesia polycarpa Maxim using ultrafiltration and dextran gel chromatography, combined with mass spectrometry to identify components with significant antioxidant activity, and screened and characterized peptides using various techniques, such as network pharmacology and molecular docking. In addition, Yasen et al. [51] isolated and purified *Cuminum cyminum* L. Seeds by 50% ethanol extraction, C18 reversed-phase column chromatography, and ion exchange chromatography for the first time, and obtained antimicrobial and antioxidant peptides. Then, the separated fractions were characterized by gel electrophoresis (SDS-PAGE) and high-pressure liquid chromatography, and the peptide components and molecular weight were determined by liquid chromatography and mass spectrometry. A novel antimicrobial peptide was prepared from cauliflower and digested and hydrolyzed with pepsin by ultrafiltration, molecular sieve chromatography, ion exchange chromatography, and mass spectrometry. The best active hydrolysates were further purified from the Changii Radix hydrolysates by ultrafiltration, size exclusion chromatography, and semi-preparative high-performance liquid chromatography [52].The peptides in the most active portion were then identified and screened for biological activity by nanoscale LC-MS/MS. The enzymolysis products from *Limnospira maxima* protein were isolated and purified with a 10 kDa range of peptides using ultra-membrane filtration, SDS-PAGE, and TLC. The resulting products showed antibacterial properties against *Escherichia coli* and *Staphylococcus aureus* [53]. In a study, the ACE inhibitory peptide HPVTGL was identified, which is derived from the protein hydrolysis product of rape (*Brassica napus* L.) bee pollen. It was obtained mainly by ultrafiltration separation and purification using preparative high-performance liquid chromatography [54].

In conclusion, the separation and purification of PDBPs is a complex process, which requires comprehensive consideration of the preparation method, separation and purification technology, and identification technology of bioactive peptides. A single separation and purification method may be limited by equipment. A combination of techniques is often used to achieve better separation of peptide mixtures, resulting in high-purity peptides as well as higher bioactivity peptides. With further advances in technology, we expect to obtain bioactive peptides from plants more efficiently, contributing to human health.

## 4. Bioactivities of PDBPs

**Figure 2:**
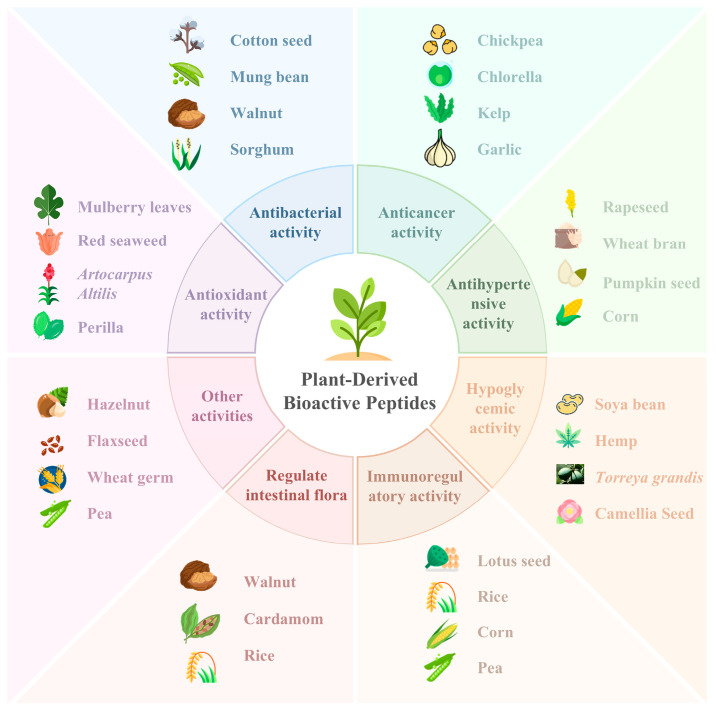
Multiple bioactivities of PDBPs.

PDBPs are hydrolyzed products obtained from plants. PDBPs have powerful functions, but their structures can be as simple as two amino acids or as complex as twenty amino acids [55]. The advantages of PDBPs are mainly focused on the two aspects of being more easily absorbed by the human body and possessing physiological functions far beyond those of amino acids. As shown in Figure 2, the biological activities of PDBPs mainly include antioxidant, anticancer, antibacterial, antihypertensive, hypoglycemic, immunomodulation, and the regulation of intestinal flora and so on.

### 4.1. Antioxidant Activity

**Figure 3:**
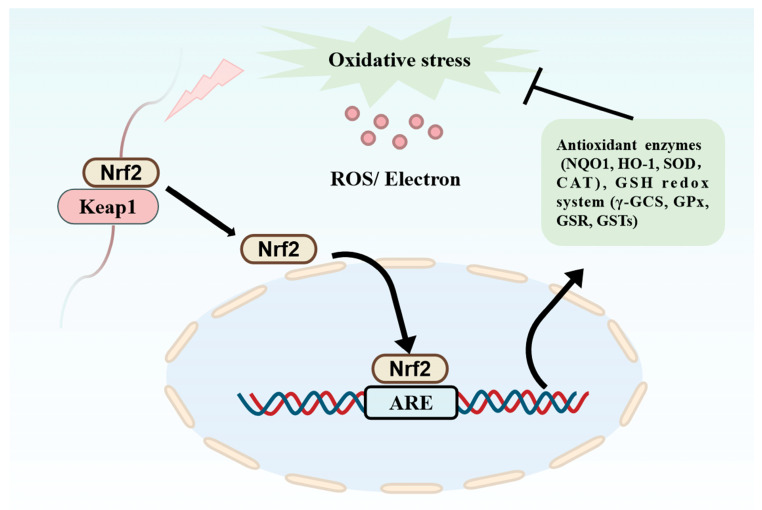
Keap1/Nrf2 signaling pathway.

Redox imbalance produces reactive oxygen species. However, excessive production of reactive oxygen species or insufficient scavenging capacity may lead to accumulation of oxidative stress, which in turn triggers inflammation, cellular damage, tissue senescence, and decreased organ function [56]. Studies have shown that natural antioxidants maintain cell health by neutralizing harmful free radicals. Furthermore, PDBPs are a good source of natural antioxidants, which can neutralize excess free radicals and stabilize their electrons, thus protecting cells from oxidative damage. They are also beneficial for preventing or treating oxidative stress-related sub-health or disease states [57]. The Keap1/Nrf2 signaling pathway has attracted much attention in studies of the antioxidant mechanisms of PDBPs (Figure 3). Currently, PDBPs are of great interest because of their high safety, good absorption, and wide array of sources. As shown in Table 2, bioactive protein hydrolysates or peptides have been obtained from different plant sources, such as seeds, nuts, leaves, and their plant by-products.

### 4.2. Antibacterial Activity

**Figure 4:**
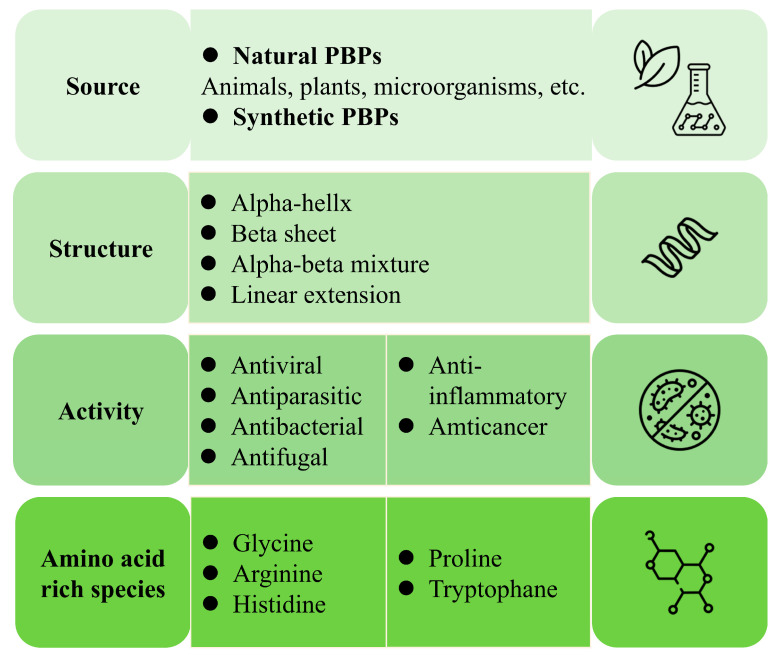
Antimicrobial peptides are classified according to different criteria.

With the proliferation of antibiotics and chemical pesticide use, there is a growing problem of resistance in flora as well as in antibiotic residues. This has become one of the factors jeopardizing public health. Therefore, there is an urgent need for alternatives to antibiotics to reduce antibiotic resistance [73]. In recent years, antimicrobial peptides have emerged as promising drug candidates against drug-resistant pathogens. Researchers have been working to find antimicrobial peptides of natural origin. Typically, antimicrobial peptides are categorized according to different criteria including their source, structure, activity, and amino acid composition (Figure 4). A variety of antimicrobial peptides have been isolated from microorganisms, insects, amphibians, plants, and mammals [74]. Among them, plants are a promising source of antimicrobial peptides. Compared with antibiotics, antimicrobial peptides of plant origin have advantages, such as low toxicity and high efficiency, as well as a broad spectrum and structural diversity. They are present in almost all areas of life as part of the innate immune system. They can fight viruses, bacteria, fungi, and even cancer cells, and are the first line of defense against invading pathogens [75]. It has been shown that bioactive peptides obtained from hydrolyzed adzuki bean and mung bean exhibited antibacterial activity against *Salmonella typhimurium* and *Staphylococcus aureus*, respectively [76]. Kong et al. [77] hydrolyzed cottonseed protein and identified three novel bacteriostatic peptides, HHRRFSLY, KFMPT, and RRLFSDY, by isolation and purification. These three peptides can achieve bacteriostatic effect by disrupting the cell membrane of *Escherichia coli*. In addition, Hu et al. [78] prepared bacteriostatic peptides from fermented walnut meal and identified four bacteriostatic peptides, FGGDSTHP, ALGGGY, YVVPW, and PLLRW. Antimicrobial peptide was obtained from sorghum spent grain hydrolysate. It can counteract the activities of two Gram-negative and three Gram-positive bacteria via the minimum inhibitory concentration method [79]. *Moringa oleifera* seed protein hydrolysates were identified as a novel antimicrobial peptide named MOp2 (HVLDTPLL), which can cause irreversible membrane damage to *Staphylococcus aureus* by increasing membrane permeability, leading to the release of the intracellular nucleotide pool. In addition, molecular docking showed that MOp2 can inhibit *Staphylococcus aureu* growth by interacting with dihydrofolate reductase and DNA lyase through hydrogen bonding and hydrophobic interactions [80].

### 4.3. Anticancer Activity

Cancer is the second leading cause of death worldwide after cardiovascular disease. However, some anticancer drugs have significant side effects and are prone to damaging normal cells [81]. Therefore, the development and utilization of natural anticancer peptides is an inevitable trend. Currently, the potential of bioactive peptides as anticancer agents of natural origin has been widely reported based on in vitro and in vivo tests. They have been demonstrated to have various anticancer effects on mature cancer cell lines, including the inhibition of cell migration, the inhibition of angiogenesis, antioxidant properties, the inhibition of cell proliferation, the induction of apoptosis, and cytotoxicity [82]. Meanwhile, bioactive peptides obtained from plant sources have received increasing attention for their potential as complementary therapies and have been promoted as promising therapeutic treatments for a variety of human diseases, especially due to their bioactivities with anticancer potential. Several studies have shown that bioactive peptides derived from a variety of plant proteins, such as those obtained from legumes, algae, garlic and rice berry, have antiproliferative and toxic effects on cancer cells, favoring cell cycle arrest and apoptosis. Mung bean protein hydrolysate, obtained by the hydrolysis of papain, has been shown to inhibit the proliferation of mouse tumor cells without the side effects of chemical anticancer drugs, and mung bean peptide promotes apoptosis in HepG2 cells and blocks the cell cycle at a lower S phase [83]. The hydrolyzed product of black soy protein was isolated and purified by ultrafiltration and chromatography. The amino acid sequence was identified as Leu/Ile-Val-Pro-Lys, which showed high cytotoxic potential against HepG2, MCF-7, and Hela cells. Molecular docking studies showed that the purified peptide efficiently bound to four apoptosis-related key proteins (XIAP, caspase-3, caspase-7, and Bcl-2) through hydrophobic interaction and hydrogen bonding [84]. It has been reported that some components of Mucuna pruriens beans peptides show high activity to protect DNA damage. 5–10 kDa exhibited significant cytotoxic activity against HepG2 and QGY-7703, and the related gene protective effects were thought to be significantly different [85]. Chickpea peptide induced S phase and G2 phase cell cycle arrest in a dose-dependent manner. DNA breakage and apoptosis were induced by downregulating Bcl-2 expression, upregulating Bax expression, and promoting caspase-3 activation [86]. Similarly, bioactive substances found in algae have effective anticancer properties. This promising and prolific source works primarily by causing apoptosis and by preventing cell division through disrupted signaling pathways. Among the identified bioactive compounds, peptides, such as VECYGPNRPQF from Chlorella, have shown significant anti-proliferative effects, especially on the gastric cancer cell line AGS, underlining their potential for further development in cancer therapy [87]. In addition, three phycocyanin-derived bioactive peptides with predicted anticancer ability were identified to significantly inhibit the growth and migration of A549, H1299, and LTEP-a2 cells. They also inhibited Akt pathway activity in NSCLC cells [88]. Another in vivo study found that LCP-3 [cyclo-(Trp-Leu-His-Val)] isolated from kelp inhibited the growth of colon cancer and induced apoptosis in cancer cells of loaded mice using an anticancer activity tracking assay [89]. Recently, scholars have identified the anticancer activity of peptides isolated from garlic against leukemia cell lines, and have also discovered a novel anticancer peptide, VKLRSLLCS. It has been shown to significantly inhibit the proliferation of MOLT-4 and K562 leukemia cell lines, and has apoptosis-inducing properties on leukemia cell lines through the anti-apoptotic Bcl-2 protein family [90]. The hydrolyzed rice bran extract had inhibitory effect on colon cancer cell line but had little effect on normal cells. Riceberry rice bran protein hydrolyzed fractions can induce apoptosis of metastatic cancer cells and senescence of non-metastatic cancer cells [91].

### 4.4. Antihypertensive Activity

**Figure 5:**
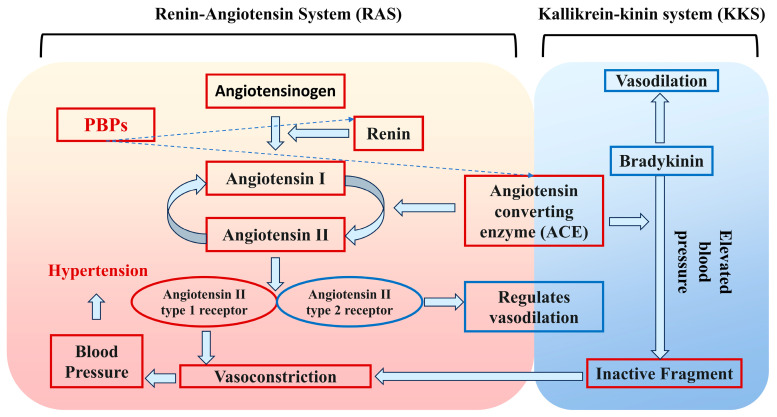
Two systems that regulate blood pressure.

Hypertension is an important global health problem affecting about one-third of the world’s adult population, and it is a high risk factor for cardiovascular diseases, such as end-stage renal disease, stroke, atherosclerosis, and myocardial infarction [92]. As shown in Figure 5, the two major systems regulating blood pressure are the renin-angiotensin system (RAS) and the kallikrein-kinin system (KKS). ACE plays an important role in the regulation of blood pressure, mainly acting on angiotensin I. It can produce angiotensin II through the RAS, which in turn inactivates bradykinin in the KKS, ultimately leading to an increase in blood pressure. Therefore, inhibition of ACE activity can play a role in lowering blood pressure [93]. At present, more studies have confirmed that specific peptides obtained by enzymatic digestion have strong ACE inhibitory activity. This has been demonstrated by the Red Alga *Acrochaetium* sp. screening of bioactive peptides. The protein hydrolysate was isolated by chromatography, and VGGSDLQAL (VL-9) was identified. The peptide VL-9 shows the ACE inhibitory activity with IC50 value 433.1 ± 1.08 µM [94]. Duan et al. [95] isolated and characterized the novel ACE inhibitory peptides FQW, FRW, and CPF from rapeseed. They showed strong ACE inhibitory activity in vitro with IC50 values of 46.84 μmol/mL, 46.30 μmol/mL, and 131.35 μmol/mL, respectively. Moreover, these peptides can interact with ACE active sites through hydrogen bonding and hydrophobic interaction. Corn gluten meal protein hydrolysate was found to not only inhibit ACE and renin activity, but also to regulate the biosynthesis and metabolism of fatty acids, sex hormones, and aldosterone through a rat model of spontaneous hypertension, thus contributing to a reduction in hypertension. In addition, Zou et al. [55] also found that wheat bran protein hydrolysates had a significant inhibitory effect on renin and ACE in rats under the condition of constructing a rat model of spontaneous hypertension [96]. Some researchers have identified some promising plant-derived raw materials for the preparation of antihypertensive peptides. SNHANQLDFHP and PVQVLASAYR were identified from pumpkin seed meal hydrolysate. The two peptides performed a protective function on EA. hy926 cells by decreasing the secretion of endothelin-1, increasing the release of nitric oxide, and regulating the ACE 2 activity [97]. *Azolla pinnata* fern protein is deeply hydrolyzed (30%) by alkaline protease to produce hydrolysates with antihypertensive (ACE inhibitory) activity [98]. Peptides extracted from water lentils [99], oil palm kernel [100], green basil leaves [101], and quinoa [102] have been found to have antihypertensive activity.

### 4.5. Hypoglycemic Activity

**Figure 6:**
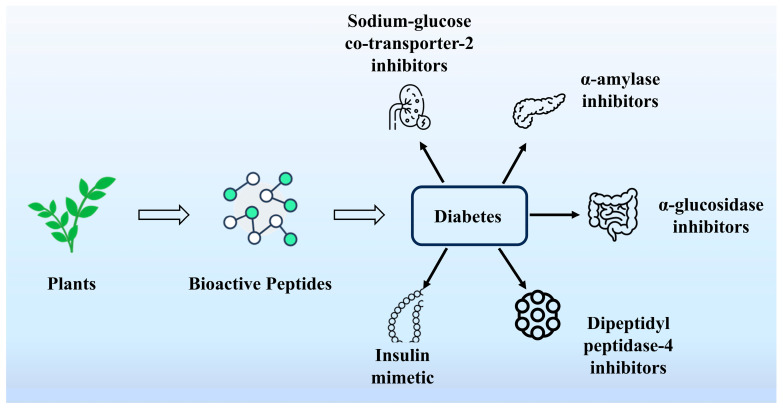
Several key enzymes that regulate blood sugar.

Diabetes mellitus is a serious and complex chronic metabolic disease characterized by elevated blood glucose due to insulin resistance or inadequate insulin secretion [103]. Recent processing technologies and human nutrition have identified plant proteins as an important source of food-derived bioactive compounds. These bioactive peptides are potential active ingredients. Among them, bioactive peptides with hypoglycemic activity can alleviate the hyperglycemic state and achieve blood glucose lowering without the help of drugs [104]. As shown in Figure 6, α-amylase, α-glucosidase, and so on are key enzymes that regulate blood glucose. Inhibiting the activity of these enzymes is considered an effective strategy for controlling diabetes. It has been shown that the peptides TGGR, SPVI, FY, and FR obtained from hemp (*Cannabis sativa* L.) protein by identification, molecular docking, and virtual screening exhibited good α-glucosidase inhibitory activities, respectively. Animal experiments showed that these peptides could regulate blood glucose and blood lipids in hyperglycemic rats [105]. Multi-enzyme hydrolyzed *Amygdalus communis* L. purified by ultrafiltration showed the best inhibitory activity of active peptide B4 against α-amylase and α-glucosidase [106]. TGPs-75 obtained from *Torreya grandis* meal peptides significantly reduced blood glucose concentrations. According to transcriptome analysis, 382 genes were significantly differentially expressed after TGPs-75 pretreatment. The main functions of these genes were related to gluconeogenesis and insulin resistance. This study suggests the possibility of using peptides from camellia seeds in camellia seed cake as hypoglycemic compounds for the prevention and treatment of diabetes [107]. Rapeseed-derived ELHQEEPL showed significant dipeptidyl peptidase-IV (DPP-IV) inhibitory activity [108]. Currently, there are also legume peptides and hydrolysates that have shown significant hypoglycemic effects by various methods [109]. For example, soybean peptides produced by alkaline protease hydrolysis exhibited the highest hypoglycemic activity by Xu et al. By analyzing their molecular weight distribution and amino acid composition, a positive correlation between the aromatic and hydrophobic amino acid content of the alkaline protein hydrolysate and the hypoglycemic activity was found [110].

### 4.6. Immunoregulatory Activity

The immune system is an autonomous defense system that prevents and controls various infectious diseases. Diseases caused by an imbalance in immunomodulation affect the body’s immune response [111]. However, many immunomodulatory drugs are not suitable for long-term or prophylactic use. Immunomodulatory peptides have been reported from various plant sources, such as soybean, lotus seed, rice, hemp, corn, pea, and others. This offers potential options for the treatment of immune-related diseases and the development of immunomodulatory therapies. It has been noted that soy peptides have multiple immunomodulatory activities on both innate and adaptive immune responses [112]. Soy peptides were prepared by hydrolyzing soy protein by alkaline protease. Furthermore, sequences of 51 peptides were identified by UPLC-MS/MS, of which 46 peptides were designated as immunomodulatory peptides. They could promote macrophage proliferation, increased cytosolic activity, and NO levels [113]. In addition, the low-molecular-weight peptide (<3 kDa) from lotus seeds had the greatest effect on the phagocytosis of RAW264.7 macrophages relative to other molecular weight peptides (>10 kDa, 5–10 kDa, 3–5 kDa). It also significantly elevated the amounts of NO, IL-6, and TNF-α secreted by peritoneal macrophages in immunosuppressed mice [114]. Immunomodulatory peptide (YGIYPR) was prepared from rice protein hydrolysate. It enhanced the proliferation of macrophage RAW 264.7 cells in the range of 12.5–100 μg/mL, and good proliferation was achieved even at the smallest doses [115]. The immunomodulatory properties of hemp protein hydrolysates (HPH) may have beneficial effects on intestinal epithelial levels [116]. Similarly, maca belongs to a group of protein-rich edible plants with immunomodulatory activity. Maca protein hydrolysate enhances phagocytosis and the secretion of NO, TNF-α, and IL-6 by RAW 264.7 cells [117]. There are also studies that have identified immunomodulatory peptides from corn proteins using human macrophage-like U937 cells [118]. In addition, pea protein hydrolysates could significantly increase the immunomodulatory activity of the macrophages by elevation of phagocytic activity and promoting the production of nitric oxide and pro-inflammatory cytokines (TNF-α and IL-6) [119].

### 4.7. Regulation of Gut Flora

**Figure 7:**
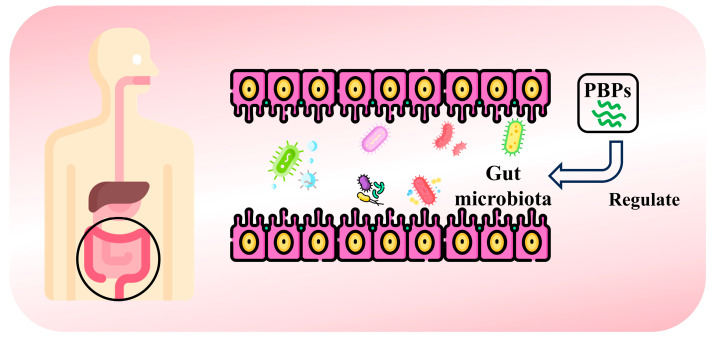
Schematic illustration of obtaining bioactive peptides from plants to regulate gut microbiota.

The various microorganisms that live in the gut form a complex, dynamically balanced intestinal flora. This intestinal barrier is an essential defense measure in the gut, providing strong protection against harmful microorganisms both internally and externally [120]. In recent years, it has been widely recognized that gut microorganisms play an important role in a variety of human diseases and are considered to be the “second genome” of the human body [121]. Studies have shown that bioactive peptides can prevent the proliferation of external bacteria and viruses, thereby maintaining the balance and stability of the gut microbiota [122]. Currently, bioactive peptides can be obtained from plants to regulate gut flora (Figure 7). Walnut protein-derived peptide LPF has protective and restorative effects on DSS-induced colitis in mice. After LPF was administered, the composition of intestinal flora in mice was continuously improved during the convalescence period of colitis. The relative abundance of beneficial bacteria increased, while the abundance of potentially harmful bacteria decreased [123]. Similarly, walnut peptide (WP) can increase the level of bacteroides and decrease the level of firmicutes in the intestinal flora of obese mice. At the same time, the abundance of the genus *Adlercreutzia* spp., which is positively associated with BMI and inflammation, decreased significantly, and *Lactobacillus* and *Butyricimonas*, which have been shown to prevent obesity and regulate intestinal microecological balance, increased significantly across the entire bacterial genus group [124]. Zhang et al. [125] analyzed the regulation and influence of cardamom selenium-containing peptides on intestinal microflora. The abundance of *Akermania*, *Firmicutes*, and *Bacteroidetes* was higher in mice treated with cardamom antibiotics containing selenium peptide, while the abundance of proteobacteria was lower. In another study, rice glutelin peptides helped to increase *Enterococcus* and *Bacteroides* in the gut, while *Streptococcus*, *Lactobacillus*, *Faecalibacterium*, *Parasutterella*, and *Turicibacter* were decreased [126]. Ginseng peptide (GP) can improve intestinal flora disorder in T2DM mice by regulating ginseng peptide abundance. *Ruminococcus* and *Bifidobacterium* are the main hypoglycemic bacteria affected by GP-H effect [127]. In addition, Li et al. [128] significantly improved the abundance and homogeneity of intestinal flora after feeding with soybean-derived peptides (SPep) for 35 days. SPep significantly promoted the growth of *Lactobacillus* and *Phascolarctobacterium*.

### 4.8. Other Biological Activities

In addition to the biological activities mentioned above, PDPBs have been found to possess anti-inflammatory activity and anti-fatigue activity, to improve neurodegenerative diseases, have anti-osteoporosis activity, have cholesterol-lowering activities, and so on. For example, hazelnut protein-derived peptide LDAPGHR has been shown to possess not only immunomodulatory activity, but also effective anti-inflammatory activity by inhibiting the production and mRNA expression of IL-1β, IL-6, and TNF-α [129]. In the weight-bearing swimming test, mice fed pea peptides were able to swim longer than the control mice [130]. LPF, GVYY, and APTLW isolated from walnut protein hydrolysate by chromatography have been reported to ameliorate LPS-induced memory deficits by normalizing inflammatory responses and oxidative stress in the brain [131]. In another in vivo experiment, wheat germ peptide ADWGGPLPH can effectively reduce the level of oxidative stress and improve microstructure and bone density in aged osteoporotic rats. In addition, ADWGGPLPH can enhance the proliferation and differentiation of osteoblasts and inhibit the differentiation of osteoclasts by regulating the OPG/RANKL/RANK/TRAF6 pathway [132]. At the same time, Yuan et al. [133] found that flaxseed peptide (FP5) with a molecular weight of less than 1 kDa can reduce cholesterol absorption and synthesis. The serum and liver cholesterol levels were significantly reduced after FP5 supplementation in rats with high cholesterol and high fat. FP5 improves the mechanism of liver cholesterol metabolism, inhibits de novo cholesterol synthesis, promotes bile acid synthesis and excretion, and inhibits bile acid reabsorption.

## 5. Structure–Activity Relationship of PDBPs

The complex biological structure and diverse functions of peptides are mainly determined by the number, composition, and arrangement of amino acids and the spatial structure of peptide chains. As shown in Table 3, the structure–activity relationship of PDBPs extracted and isolated from plants were identified.

### 5.1. Molecular Weight

It is well known that extensive hydrolysis produces small peptides (1–3 kDa) that are more likely to be bioavailable and biologically active. Chen et al. [148] explored the effect of molecular weight on the antioxidant capacity of rice protein hydrolysate. Corn gluten meal hydrolysate was obtained by hydrolysis using a combination of alcalase, flavorzyme, and protamex, and the hydrolysate was analyzed for antioxidant activity after further fractionation. Corn peptides with a molecular weight less than 1 kDa showed excellent antioxidant activity [149]. In particular, peptides with molecular weights less than 3 kDa showed better ACE inhibition compared to larger peptides. A low-molecular-weight (LMW) fraction (<3 kDa) was extracted from *Olea europaea* (cv. Farga). The LMW grade has a strong ACE inhibitory activity [150]. In addition, low-molecular-weight peptides were prepared from soybean dregs using high-pressure homogenization-assisted dual enzymes, the structure of which resulted in changes only in the hydrogen bonding between peptide chains. It promoted cellular phagocytosis, NO levels, and the release of cytokines IL-6, IFN-γ, and TNF-α [151]. Another study found that most of the peptide fragments of low-molecular-weight peptides extracted from red macroalgae possessed different levels of antioxidant, antimicrobial, anti-ACE, and anti-DPP-IV inhibitory activities [152]. A preparation of oat peptides using in vitro simulated digestion produced low-molecular-weight antimicrobial peptides that ameliorated colitis in rats [153].

### 5.2. Amino Acid Composition and Sequence

In terms of amino acid composition, the biological activity of PDBPs is related to the proportion of acidic and basic amino acids, hydrophobic amino acids, and aromatic amino acids that they contain [154]. Take antioxidant peptides as an example: a recent study has shown that chemical groups on amino acid residues are related to the antioxidant activity of peptides. Phenolic hydroxyl groups in Tyr, indole groups in Trp, and imidazole groups in His of peptides may increase their interaction with free radicals [155]. In addition, aromatic amino acid residues (Tyr, Trp, and Phe) have been reported to play a key role in the antioxidant activity of peptides [156]. Phenolic hydroxyl groups in tyrosine and nitrogen-hydrogen bonds in the indole ring of tryptophan of corn gluten meal hydrolysate are key sites for antioxidant activity. These findings may explain the effective chemical antioxidant activity [157]. Cottonseed is rich in acidic and basic amino acids, as well as aromatic and hydrophobic amino acids, which may contribute to its antioxidant capacity [158].

In amino acid sequences, peptides with similar chain lengths exhibit different functional activities according to their *C*-terminal and *N*-terminal amino acid sequences. Molecular docking studies of the three peptides of quinoa protein hydrolyzate revealed that the presence of specific amino acids in the peptide sequence (Pro, Phe, and Arg at the *C*-terminal, and Asn at the *N*-terminal) may contribute to the interaction between ACE and peptides [159]. Another study found that the *C*-terminal extension of SbGPRP1 from sorghum bicolor could act as an antimicrobial peptide by targeting bacterial outer membrane proteins [160].

### 5.3. Secondary Structure

The secondary structure usually refers to the spatial direction of the protein peptide chain along the backbone of the main chain, the regular cyclic arrangement, or a section of the peptide chain of the local spatial structure. That is, the relative spatial coiling and folding positions of the main chain of the peptide chain or the backbone atoms of a section of the peptide chain, which does not involve the conformation of the side chain of amino acid residues [161]. The common secondary structures are α-helix, β-fold, β-corner, and random curling. The stepwise enzymatic hydrolysis of coix seed prolamins promoted the destruction of the secondary structure of the hydrolyzed products, enhanced the β-angle structure, and increased the DPP-IV inhibitory activity [162]. Dual enzyme digestion products of walnut dreg showed an increased β-fold content and an altered bitter flavor intensity [31]. Habinshuti et al. have shown that the compactness of the peptide structure affects its antioxidant properties [163]. Similarly, Tia et al. found a decrease in β-folding from 33.74% to 15.64% and an increase in β-turning angle from 30.93% to 47.36% after the hydrolysis of malt albumin by ultrasound-assisted papain. At the same time, DPPH could reach 82.29% [164]. In addition, Liang et al. showed that changes in the secondary structure of active peptides directly affect the antioxidant activity of peptides [165]. Hydrolyzed soybean meal hydrolysates showed an increase in the number of β-turns and a decrease in α-helices and exhibited high antioxidant and ACE inhibitory activities [166]. Mulberry leaf protein hydrolysate, which consists mainly of disordered convolutions and β-folds, has antioxidant activity [167].

## 6. Application of PDBPs in Chronic Diseases

The full name of chronic disease is chronic non-communicable disease, and does not specifically refers to a disease, but rather refers to a class of diseases which have a long course and complex causes, and which cause health damage and serious social harm [168]. There is a lack of evidence of an exact infectious biological cause for such diseases; the etiology is complex, and some diseases have not been fully identified. At present, the complexity of chronic diseases poses a great challenge to our understanding of such diseases [169]. Cardiovascular and cerebrovascular diseases, diabetes, cancer, mental disorders, and other diseases are increasing and affecting people’s quality of life [170,171,172]. Many of these diseases are linked to lifestyle, environment, diet, or genetic factors. As a result, the scientific community’s study for new drugs and functional foods that can combat or prevent chronic diseases has increased over the past few decades [173]. Some natural resources are particularly attractive when environmental, cost, and renewable factors are taken into account [174].

In the context of the great popularity of plant products, PDBPs and their derived peptides stand out as promising bioactive ingredients. PDBPs can be produced from plant proteins by a series of methods. In addition, compared with animal-derived bioactive peptides, PDBPs also have advantages in human health [175]. More and more attention has been paid to their advantages, such as their non-toxic nature, low cost, wide availability, and diverse functions. Studies have shown that PDBPs can fight cardiovascular disease, type 2 diabetes, and some cancers, among others [176]. Additionally, the potential for using PDBPs to develop functional foods and drugs is huge. As far as we know, many antihypertensive drugs can cause side effects, such as dizziness [177]. As a result, the search for antihypertensive biopeptides from plants has increased. Wang et al. [178] evaluated the safety and antihypertensive activity of rapeseed peptides. The potential synergies with captopril were also discussed. In in vitro experiments, it was found that their synergistic effect further increased the serum levels of NO and endothelial nitric oxide synthase in rats. In another study, Lai et al. [179] optimized the extraction of green tea residue by alkaline extraction combined with enzymatic hydrolysis. Under optimized hydrolysis conditions, the ACE inhibition rate of the product was found to be 77.00%, and the molecular weight distribution of the polypeptide in the hydrolyzed product was widely in the range of 45.0–1.2 kDa. At the same time, in vivo experimentation also confirmed the good blood pressure lowering activity of this peptide. ACE inhibitory peptides were purified and identified from cashews, among which FETISFK showed the highest ACE inhibitory rate (91.04 ± 0.31%). It also reduced the expression of angiotensin II and angiotensin II type 1 receptors [180]. Type 2 diabetes is the dominant form of diabetes, accounting for more than 90% of the patient population. For clinical treatment, α-glucosidase inhibitors are a type of T2DM alternative therapy to regulate postprandial blood glucose levels [181]. At present, α-glucosidase inhibitory peptides have been isolated and identified from many natural sources. Three novel potential bioactive peptides (RWPFFAFM, AAGRLPGY, and VVRDFHNA) were screened from mulberry leaf proteins to inhibit α-glucosidase [182]. In addition, the α-glucosidase inhibitory activity of WGPs was identified with an IC 50 value of 6.87 mg/mL. LDLQR, AGGFR, and LDNFR were synthesized for further identification. The results of molecular docking and amino acid composition analyses showed that the high content of *C*-terminal Arg residues in the peptide may be the essential reason for its inhibition of α-glucosidase activity [183]. Recently, a highly active α-glucosidase inhibitory peptide with the amino acid sequence KETTTIVR was identified from Moringa oleifera seed protein hydrolysates, which was found to be a amphiphilic peptide with a β-corner structure [184]. PDBPs also contain anticancer peptides that help to improve and prevent the occurrence of a range of chronic cancers, more in line with the new generation of consumers’ pursuit of a healthy diet [185]. For example, cyclic peptides isolated from marine cyanobacteria, such as *Urumamide*, exhibited low proliferative inhibitory activity on human cancer cells [186]. Rapeseed peptide inhibited cell proliferation by regulating the P53 signaling pathway, inducing G0/G1 phase arrest, and the mitochondrial apoptosis pathway [187]. Recently, a peptide has been isolated that regulates the proliferation and invasion of non-small cell lung cancer cells. This polypeptide is derived from Bryopsis plumosa. Kim et al. obtained anticancer peptides through the experimental steps of peptide design, synthesis, and purification. In vivo experiments, peptides can effectively inhibit the metastasis of tumor xenografts in zebrafish embryos [188]. The most studied peptide from legumes is lunasin. Over the past decade, it has been identified that it exerts its anticancer action through different pathways, including regulating cell growth and apoptosis, epigenetic mechanisms, and blocking the transformation of normal cells. Diego Luna-Vital also summarized that different legumes have been used to produce peptides with anti-gastrointestinal cancer potential, especially soybeans, mung beans, lentils, chickpeas, and common beans. Most studies have focused on evaluating the anti-proliferation effects of plant-bioderived peptides on gastrointestinal cancer cells in vitro [189]. In addition, the isolation and relative purification of bioactive peptides from *Achillea eriophora* showed that the peptide mixture inhibited the growth of MCF-7 cancer cell lines, and also showed DPPH radical scavenging activity and the inhibition of copper ion reduction [190].

Currently, many PDBP in vitro cell experiments have shown a variety of activities that have been fully confirmed. However, studies in vitro have been very limited. Their safety needs to be further explained. Therefore, it is necessary to study PDBPs in vivo.

## 7. Limitations

The research related to PDBPs is gradually increasing, but PDBPs are not used in clinical and food and pharmaceutical applications on a large scale. There are many challenges in the development of PDBPs, mainly due to the following reasons. The first is the difficulties involved in in the preparation of PDBPs. The PDBPs obtained by different preparation methods, either enzymatic or fermentation, are structurally different and the peptides obtained cannot be determined. Considering the cost, it is impossible to select the conditions of enzymatic digestion or fermentation in a targeted manner. Secondly, various factors, such as time, temperature, and pH, can be a stumbling block to the preparation of PDBPs. In addition, how to ensure the yield of PDBPs is also an important indicator that can be successfully applied to the clinic. At present, PDBPs mostly stay in in vitro experiments. In the reviewing related literature, this review found that there are relatively few in vivo studies on PDBPs compared to in vitro studies. However, the development of chronic diseases in humans is very complex, and further animal studies and randomized clinical trials are needed to further explore the pathogenesis and therapeutic mechanism of these diseases. In the future, these limitations should be deeply explored and overcome in order to realize a wider application of PDBPs in the prevention and treatment of chronic diseases.

## 8. Conclusions

PDBPs has attracted attention in many fields because of their diverse biological activities, great potential, and wide application prospects. By promoting PDBPs, we can reduce our dependence on animals, reduce greenhouse gas emissions and water consumption, and protect the ecological environment. At the same time, the promotion of PDBPs can bring about healthier and more environmentally friendly food choices for humans. The current preparation methods (enzymatic hydrolysis and microbial fermentation) have been successfully applied to the extraction of active peptides, but still need further extraction and separation. In addition, the signal pathway of how PDBPs exert their biological activity through the body metabolism, as well as their potential targets and functional mechanisms, still need to be further explored. Therefore, future research should focus more on the prevention and treatment of human chronic diseases. This is of great significance for understanding the targeted action mechanism of peptides, optimizing their biological activity, and accurately designing functional foods or drugs.

## Figures and Tables

**Table 1:** Advantages and disadvantages of two preparation methods.

Preparation Method	Advantages	Disadvantages
Enzymatic hydrolysis	No toxic substances are produced Non-destructive amino acid High catalytic efficiency High specificity	High cost
Microbial fermentation	High efficiency Low cost	Difficult to control Susceptible to microbial contamination Difficult separation and purification

**Table 2:** Antioxidant activity and effects of PDBPs.

Plant Origins	Sequence/Name	Major Findings	Reference
Pea Protein	YLVN EEHLCFR TFY3	Through computer simulation of molecular docking, it was found that the anti-oxidation mechanism may be highly related to the activation of the Keap1-Nrf2 pathway by occupying the Keap1-Nrf2 binding site.	[58]
Mulberry leaves	EGDAGAQGPPGPAGPAGER RPGPSPGVGAPGK	↓ ROS ↑ SOD and CAT Both peptides are found to exert protective effects against H_2_O_2_-induced chromatin damage and cell apoptosis.	[59]
Red seaweed (*Palmaria palmata*)	SLLYSDITRPGGNMYTTR (SR18)	The SR18 peptide was found to have extremely high ROS scavenging activity and high ferric reducing ability and may be partially driven by the amino acids Tyr, Asn, and Met.	[60]
*Artocarpus altilis* (Parkinson) Fosberg	AAPPH	The different enzymatic hydrolysis products of the hydrolysis had a higher Fe^2+^-chelating capacity, DPPH scavenging capacity, and hydrogen peroxide scavenging capacity.	[61]
Rice bran	AFDEGPWPK	AFDEGPWPK has the ability to scavenge ORAC and DPPH free radicals, and it can enter the binding pocket of the Kelch structural domain and activate the Keap1/Nrf2/HO-1 pathway.	[27]
Pea	Pea-derived peptides (PPs)	↓ SOD, GR, GSH, GSSG, and ROS	[62]
Sacha inchi (*Plukenetia volubilis* L.)	SIH20B AAGALKKFL LGVKFKGGL	Antioxidant capacity (DPPH free radical chelating capacity, ferric ion reducing antioxidant capacity, and β-carotene-linoleic acid assay)	[63]
Perilla seed meal	NFF and PMR perilla seed peptides (PSP)	↓ ROS	[64]
Potato	IFGPM, IDGGGI, HGPHIF, VDDDKDFIPF, LVTVDDDKD, VVTGGKVGNEND, potato protein hydrolysates (PPH)	Fe^2+^-chelating activity (55.33 µg EDTA/mL), ·OH scavenging activity (230.05 µg Vc/mL), and oxygen radical absorbance capacity (82.24 µg TE/mL)	[65]
Defatted walnut meal	AASCDQ	AQ exhibited strong antioxidant activity, which demonstrated significant scavenging ability against DPPH (79.40%), superoxide anion (81.00%), and ABTS (67.09%) radicals.	[66]
Cyperus (*Cyperus esculentus* L.)	SFRWQ	↑ SOD, CAT, ↓ ROS, TNF-α, IL-6	[67]
Coix seed (*Coix lacryma-jobi* L.)	FFDR	↑ GSH, CAT, SOD, GSH-Px ↓ GSSG, MDA	[68]
Wheat gluten	LY, PY, YQ, APSY, RGGY	↑ SOD, CAT, GSH-Px, GSH ↓ ROS, MDA	[69]
*Moringa oleifera* leaves	LALPVYN	↑ CAT, GSH-Px, SOD ↓ MDA, ROS	[70]
Soybean	Leu-Ser-Trp (LSW)	Inhibited oxidative stress and reduced superoxide and malondialdehyde levels	[71]
Walnut	HGEPGQQQR VAPFPEVFGK HNVADPQR	Excellent cellular antioxidant activity	[72]

Reactive oxygen species (ROS), superoxide dismutase (SOD), catalase (CAT), glutathione peroxidase (GSH -Px), glutathione (GSH and GSSG), glutathione reductase (GR), 1,1-diphenyl-2-picrylhydrazyl (DPPH), 2,2′-Azinobis-(3-ethylbenzthiazoline-6-sulphonate) (ABTS), oxygen radical absorbance capacity (ORAC).

**Table 3:** The structure–activity relationship of PDBPs obtained from plants.

Functional Activity	Plant Origins	Molecular Characteristics	Structure–Activity Relationship	Reference
Antioxidant peptides	Pine nut (*Pinus koraiensis* Sieb. et Zucc.)	WYSGK	The presence of Ser increases its beta-pleated sheet content, and the active hydrogen atoms produce a chemical shift.	[134]
Antioxidant peptides	Watermelon seed	RDPEER (P1)	The active site of P1 is located at C_6_H_14_ on Arg. P1 can bind to DPPH/ABTS through hydrogen bonding and hydrophobic interaction.	[135]
Antioxidant peptides	Walnut (*Juglans mandshurica* Maxim.)	Walnut protein hydrolysate (<3 kDa) and peptides KGHLFPN	The tendency of the layer secondary structure to be randomly curled during digestion and the increase in active hydrogen content are favorable conditions for improving its antioxidant capacity.	[136]
Antioxidant peptides	Tartary buckwheat (*Fagopyrum tataricum* (L.) Gaertn)	CTGFVAVR	CR-8 can enhance the antioxidant capacity of damaged cells by interfering with multiple metabolic pathways. This is associated with hydrophobic amino acids, *N*-terminal cysteine (Cys), and others.	[137]
Immunomodulatory peptides	Rice (*Oryza sativa* L.)	11–20 amino acids	S, R, D, E, and T amino acids readily form hydrogen bonds with MHC-II molecules, thereby enhancing innate and adaptive immunity.	[138]
Immunomodulatory peptides	Soybean (*Glycine max* L.)	EKPQQQSSRRGS	EKPQQQQSSRRGS increases phagocytic activity of mouse spleen macrophages and also induces macrophage M1 polarization.	[139]
Immunomodulatory peptides	Sunflower seed (*Helianthus annuus* L.)	MVWGP	MVWGP is the most potent immunomodulatory peptide in all cellular assays, which is attributed to the presence of Met residues.	[140]
Antimicrobial peptides	Genus *Ulva* (Ulvophyceae, Chlorophyta)	HAVY RDRF	HAVY has a large number of hydrogen bonds as an antimicrobial agent and has shown higher antimicrobial efficacy than RDRF in in vitro validation.	[141]
Antimicrobial peptides	*Moringa oleifera*	HVLDTPLL	HVLDTPLL inhibited *S. aureus* growth by interacting with dihydrofolate reductase and DNA gyrase through hydrogen bonding and hydrophobic interactions.	[80]
Antimicrobial peptides	Chia seeds (*Salvia hispanica* L.)	<1 kDa	Significant inhibition effect was reported against *Listeria monocytogenes* for components with molecular weight < 1 kDa.	[142]
Anticancer peptides	Walnuts (*Juglans regia* L.)	<1 kDa	The <1 kDa molecular weight fraction (WPH-M1) exhibited more significant inhibition of HCT116 cell proliferation and induction of apoptosis than other fractions.	[143]
Anticancer peptides	Amaranth seed (*Amaranthus caudutus* L.)	Heat denaturation	Bioactive peptides from amaranth seed protein hydrolysates induced apoptosis and antimigratory effects in breast cancer cells.	[144]
Anticancer peptides	Corn gluten meal	3–5 kDa	The fraction 3–5 kDa effectively inhibited the growth of HepG2 cancer cells.	[145]
Antidiabetic peptides	Highland barley	F-3 (DH-23.86%)	F-3 exhibited strong antiglycation activity, effectively suppressed the non-fluorescent AGE (CML) and the fructosamine level.	[146]
Antidiabetic peptides	Hemp (*Cannabis sativa* L.) seed meal	99% of hydrolysate peptides have molecular weights < 5 kDa, 53.95% are 0.5–1 kDa	The peptides inhibited both intracellular disaccharidase and the transport of glucose at different concentrations.	[147]

## References

[1] Thakur S., Pandey A.K., Verma K., Shrivastava A., Singh N. (2024). Plant-based protein as an alternative to animal proteins: A review of sources, extraction methods and applications. Int. J. Food Sci. Technol..

[2] Päivärinta E., Itkonen S.T., Pellinen T., Lehtovirta M., Erkkola M., Pajari A.-M. (2020). Replacing Animal-Based Proteins with Plant-Based Proteins Changes the Composition of a Whole Nordic Diet—A Randomised Clinical Trial in Healthy Finnish Adults. Nutrients.

[3] Görgüç A., Gençdağ E., Yılmaz F.M. (2020). Bioactive peptides derived from plant origin by-products: Biological activities and techno-functional utilizations in food developments—A review. Food Res. Int..

[4] Sosalagere C., Adesegun Kehinde B., Sharma P. (2022). Isolation and functionalities of bioactive peptides from fruits and vegetables: A reviews. Food Chem..

[5] Aderinola T.A., Duodu K.G. (2022). Production, health-promoting properties and characterization of bioactive peptides from cereal and legume grains. BioFactors.

[6] Li X., Guo M., Chi J., Ma J. (2020). Bioactive Peptides from Walnut Residue Protein. Molecules.

[7] Nourmohammadi E., Sadeghi Mahoonak A. (2018). Health Implications of Bioactive Peptides: A Review. Int. J. Vitam. Nutr. Res..

[8] Liu Y.Q., Strappe P., Shang W.T., Zhou Z.K. (2019). Functional peptides derived from rice bran proteins. Crit. Rev. Food Sci. Nutr..

[9] Liu J., Zhu X., Kim S.J., Zhang W. (2016). Antimycin-type depsipeptides: Discovery, biosynthesis, chemical synthesis, and bioactivities. Nat. Prod. Rep..

[10] Cruz-Casas D.E., Aguilar C.N., Ascacio-Valdés J.A., Rodríguez-Herrera R., Chávez-González M.L., Flores-Gallegos A.C. (2021). Enzymatic hydrolysis and microbial fermentation: The most favorable biotechnological methods for the release of bioactive peptides. Food Chem. Mol. Sci..

[11] Karami Z., Akbari-adergani B. (2019). Bioactive food derived peptides: A review on correlation between structure of bioactive peptides and their functional properties. J. Food Sci. Technol..

[12] Nasri M., Toldrá F. (2017). Chapter Four—Protein Hydrolysates and Biopeptides: Production, Biological Activities, and Applications in Foods and Health Benefits. A Review. Advances in Food and Nutrition Research.

[13] Sun X., Acquah C., Aluko R.E., Udenigwe C.C. (2020). Considering food matrix and gastrointestinal effects in enhancing bioactive peptide absorption and bioavailability. J. Funct. Foods.

[14] Liu W., Chen X., Li H., Zhang J., An J., Liu X. (2022). Anti-Inflammatory Function of Plant-Derived Bioactive Peptides: A Review. Foods.

[15] Kannan A., Hettiarachchy N.S., Lay J.O., Liyanage R. (2010). Human cancer cell proliferation inhibition by a pentapeptide isolated and characterized from rice bran. Peptides.

[16] Cicero A.F.G., Fogacci F., Colletti A. (2017). Potential role of bioactive peptides in prevention and treatment of chronic diseases: A narrative review. Br. J. Pharmacol..

[17] Duffuler P., Bhullar K.S., de Campos Zani S.C., Wu J. (2022). Bioactive Peptides: From Basic Research to Clinical Trials and Commercialization. J. Agric. Food Chem..

[18] Lins M., Puppin Zandonadi R., Raposo A., Ginani V.C. (2021). Food Waste on Foodservice: An Overview through the Perspective of Sustainable Dimensions. Foods.

[19] Memarpoor-Yazdi M., Karbalaei-Heidari H.R., Khajeh K. (2017). Production of the renewable extremophile lipase: Valuable biocatalyst with potential usage in food industry. Food Bioprod. Process..

[20] Courrol D.d.S., Silva C.C.F.d., Prado L.G., Chura-Chambi R.M., Morganti L., de Souza G.O., Heinemann M.B., Isaac L., Conte F.P., Portaro F.C.V. (2022). Leptolysin, a Leptospira secreted metalloprotease of the pappalysin family with broad-spectrum activity. Front. Cell. Infect. Microbiol..

[21] An G., Wei M., Wang Z., Zhao X., Li X., He Z. (2022). Effects of enzymolysis method on the preparation of peptides from wheat flour. Food Biosci..

[22] Xiang H., Waterhouse D.-S., Liu P., Waterhouse G.I.N., Li J., Cui C. (2020). Pancreatic lipase-inhibiting protein hydrolysate and peptides from seabuckthorn seed meal: Preparation optimization and inhibitory mechanism. LWT.

[23] Song W., Kong X., Hua Y., Chen Y., Zhang C., Chen Y. (2020). Identification of antibacterial peptides generated from enzymatic hydrolysis of cottonseed proteins. LWT.

[24] Chen L., Li D., Zhu C., Rong Y., Zeng W. (2021). Characterisation of antioxidant peptides from enzymatic hydrolysate of golden melon seeds protein. Int. J. Food Sci. Technol..

[25] Tian S., Du K., Yan F., Li Y. (2022). Microwave-assisted enzymatic hydrolysis of wheat germ albumin to prepare polypeptides and influence on physical and chemical properties. Food Chem..

[26] Xu X., Qiao Y., Shi B., Dia V.P. (2021). Alcalase and bromelain hydrolysis affected physicochemical and functional properties and biological activities of legume proteins. Food Struct..

[27] Ren L.-K., Fan J., Yang Y., Liu X.-F., Wang B., Bian X., Wang D.-F., Xu Y., Liu B.-X., Zhu P.-Y. (2023). Identification, in silico selection, and mechanism study of novel antioxidant peptides derived from the rice bran protein hydrolysates. Food Chem..

[28] Liu F.-F., Li Y.-Q., Wang C.-Y., Liang Y., Zhao X.-Z., He J.-X., Mo H.-Z. (2022). Physicochemical, functional and antioxidant properties of mung bean protein enzymatic hydrolysates. Food Chem..

[29] Garcia-Vaquero M., Mora L., Hayes M. (2019). In Vitro and In Silico Approaches to Generating and Identifying Angiotensin-Converting Enzyme I Inhibitory Peptides from Green Macroalga Ulva lactuca. Mar. Drugs.

[30] Lu D., Peng M., Yu M., Jiang B., Wu H., Chen J. (2021). Effect of Enzymatic Hydrolysis on the Zinc Binding Capacity and in vitro Gastrointestinal Stability of Peptides Derived From Pumpkin (*Cucurbita pepo* L.) Seeds. Front. Nutr..

[31] Shen Y., Fang L., Liu C., Wang J., Wu D., Zeng Q., Leng Y., Min W. (2024). Effect of bi-enzyme hydrolysis on the properties and composition of hydrolysates of Manchurian walnut dreg protein. Food Chem..

[32] Ozón B., Cotabarren J., Valicenti T., Graciela Parisi M., David Obregón W. (2022). Chia expeller: A promising source of antioxidant, antihypertensive and antithrombotic peptides produced by enzymatic hydrolysis with Alcalase and Flavourzyme. Food Chem..

[33] Chen L., Cheng F., Chen H., Shu G. (2024). Preparation and identification of novel angiotensin-I-converting enzyme inhibitory peptides from Moringa oleifera leaf. LWT.

[34] Cao M., Li W., Li H., Zhang J., Liu Y., Liu X. (2022). Antioxidant and ACE inhibitory activities of peptides prepared from adzuki bean by semi-solid enzymatic hydrolysis. Food Biosci..

[35] Liu W., Yu S., Han Y., Chen L., An J., Li H., Liu X. (2024). Systematic sequence characterization of enzymatic-derived soybean peptides for precision enhancement of anti-inflammatory properties. Food Biosci..

[36] Rezvankhah A., Yarmand M.S., Ghanbarzadeh B., Mirzaee H. (2021). Characterization of bioactive peptides produced from green lentil (*Lens culinaris*) seed protein concentrate using Alcalase and Flavourzyme in single and sequential hydrolysis. J. Food Process. Preserv..

[37] Goyal N., Hajare S.N., Gautam S. (2023). Release of an encrypted, highly potent ACE-inhibitory peptide by enzymatic hydrolysis of moth bean (Vigna aconitifolia) protein. Front. Nutr..

[38] Gao Y.-L., Wang C.-S., Zhu Q.-H., Qian G.-Y. (2013). Optimization of Solid-State Fermentation with Lactobacillus brevis and Aspergillus oryzae for Trypsin Inhibitor Degradation in Soybean Meal. J. Integr. Agric..

[39] Razavizadeh S., Alencikiene G., Vaiciulyte-Funk L., Ertbjerg P., Salaseviciene A. (2022). Utilization of fermented and enzymatically hydrolyzed soy press cake as ingredient for meat analogues. LWT.

[40] Babini E., Taneyo-Saa D.L., Tassoni A., Ferri M., Kraft A., Grän-Heedfeld J., Bretz K., Roda A., Michelini E., Calabretta M.M. (2020). Microbial Fermentation of Industrial Rice-Starch Byproduct as Valuable Source of Peptide Fractions with Health-Related Activity. Microorganisms.

[41] Chen Y., Liu W., Xue J., Yang J., Chen X., Shao Y., Kwok L.-y., Bilige M., Mang L., Zhang H. (2014). Angiotensin-converting enzyme inhibitory activity of Lactobacillus helveticus strains from traditional fermented dairy foods and antihypertensive effect of fermented milk of strain H9. J. Dairy Sci..

[42] Tonini S., Tlais A.Z.A., Galli B.D., Helal A., Tagliazucchi D., Filannino P., Zannini E., Gobbetti M., Di Cagno R. (2024). Lentils protein isolate as a fermenting substrate for the production of bioactive peptides by lactic acid bacteria and neglected yeast species. Microb. Biotechnol..

[43] Marulo S., De Caro S., Nitride C., Di Renzo T., Di Stasio L., Ferranti P., Reale A., Mamone G. (2024). Bioactive peptides released by lactic acid bacteria fermented pistachio beverages. Food Biosci..

[44] Ma J., Su K., Chen M., Wang S. (2023). Study on the antioxidant activity of peptides from soybean meal by fermentation based on the chemical method and AAPH-induced oxidative stress. Food Sci. Nutr..

[45] Huang W., Xu H., Pan J., Dai C., Mintah B.K., Dabbour M., Zhou R., He R., Ma H. (2022). Mixed-Strain Fermentation Conditions Screening of Polypeptides from Rapeseed Meal and the Microbial Diversity Analysis by High-Throughput Sequencing. Foods.

[46] Chirinos R., Pedreschi R., Campos D. (2020). Enzyme-assisted hydrolysates from sacha inchi (Plukenetia volubilis) protein with in vitro antioxidant and antihypertensive properties. J. Food Process. Preserv..

[47] Wang Y., Huang Q., Kong D., Xu P. (2018). Production and Functionality of Food-derived Bioactive Peptides: A Review. Mini-Rev. Med. Chem..

[48] Alavi F., Ciftci O.N. (2023). Purification and fractionation of bioactive peptides through membrane filtration: A critical and application review. Trends Food Sci. Technol..

[49] Agyei D., Ongkudon C.M., Wei C.Y., Chan A.S., Danquah M.K. (2016). Bioprocess challenges to the isolation and purification of bioactive peptides. Food Bioprod. Process..

[50] Dou L., Zhang Z., Yang W., Chen Y., Luo K., Kan J. (2024). Separation and purification of antioxidant peptides from Idesia polycarpa Maxim. cake meal and study of conformational relationship between them. Food Sci. Nutr..

[51] Mijiti Y., Wali A., Jian Y., Rozi P., Yili A., Aisa H.A. (2018). Isolation Purification and Characterization of Antimicrobial Peptides from *Cuminum cyminum* L. Seeds. Int. J. Pept. Res. Ther..

[52] Liu L., Chen J., Li X. (2021). Novel peptides with α-glucosidase inhibitory activity from Changii Radix hydrolysates. Process Biochem..

[53] Sathya R., MubarakAli D., Mehboob Nousheen M.G., Vasimalai N., Thajuddin N., Kim J.-W. (2022). An Investigation of Pepsin Hydrolysate of Short Antibacterial Peptides Derived from *Limnospira* Sp. Appl. Biochem. Biotechnol..

[54] Zhu S., Wang S., Wang L., Huang D., Chen S. (2021). Identification and characterization of an angiotensin-I converting enzyme inhibitory peptide from enzymatic hydrolysate of rape (*Brassica napus* L.) bee pollen. LWT.

[55] Yuan H., Luo Z., Ban Z., Reiter R.J., Ma Q., Liang Z., Yang M., Li X., Li L. (2022). Bioactive peptides of plant origin: Distribution, functionality, and evidence of benefits in food and health. Food Funct..

[56] Tirichen H., Yaigoub H., Xu W., Wu C., Li R., Li Y. (2021). Mitochondrial Reactive Oxygen Species and Their Contribution in Chronic Kidney Disease Progression Through Oxidative Stress. Front. Physiol..

[57] Wong F.-C., Xiao J., Wang S., Ee K.-Y., Chai T.-T. (2020). Advances on the antioxidant peptides from edible plant sources. Trends Food Sci. Technol..

[58] Zhao D., Liu X. (2023). Purification, Identification and Evaluation of Antioxidant Peptides from Pea Protein Hydrolysates. Molecules.

[59] Zhou Y., Zhang R., Wang J., Tong Y., Zhang J., Li Z., Zhang H., Abbas Z., Si D., Wei X. (2024). Isolation, Characterization, and Functional Properties of Antioxidant Peptides from Mulberry Leaf Enzymatic Hydrolysates. Antioxidants.

[60] Liboureau P., Pampanin D.M. (2024). Effects of vegetative propagation on protein content and bioactivity of the red seaweed *Palmaria palmata*. Food Chem..

[61] Dada S.O., Ehie G.C., Osukoya O.A., Anadozie S.O., Adewale O.B., Kuku A. (2023). In vitro antioxidant and anti-inflammatory properties of *Artocarpus altilis* (Parkinson) Fosberg (seedless breadfruit) fruit pulp protein hydrolysates. Sci. Rep..

[62] Gao B., Cui C., Yan F., Li N., Sun X., Wang F., Wang C. (2024). In Vitro Protective Effect of Pea-Derived Peptides (PPs) via the Keap1/Nrf2 Signaling Pathway on Alpha-Gliadin-Sensitizing Peptide Induced Cacao-2 Cells. Mol. Nutr. Food Res..

[63] Lemus-Conejo A., Villanueva-Lazo A., Martin M.E., Millan F., Millan-Linares M.C. (2024). Sacha Inchi (*Plukenetia volubilis* L.) Protein Hydrolysate as a New Ingredient of Functional Foods. Foods.

[64] Wang L., Qu L., He B. (2024). Preparation, identification and molecular docking of two novel anti-aging peptides from perilla seed. Heliyon.

[65] Liu H., Sun H.-N., Zhang M., Mu T.-H., Khan N.M. (2023). Production, identification and characterization of antioxidant peptides from potato protein by energy-divergent and gathered ultrasound assisted enzymatic hydrolysis. Food Chem..

[66] Gao F., Zhang Z., Xue N., Ma Y., Jiao J., Wang C., Zhang K., Lin Y., Li S., Guo Z. (2024). Identification of a novel oligopeptide from defatted walnut meal hydrolysate as a potential neuroprotective agent. Food Funct..

[67] Ma C., Wu X. (2024). Cyperus peptide SFRWQ inhibits oxidation and inflammation in RAW264.7 cell model. Int. J. Biol. Macromol..

[68] Igbokwe C.J., Feng Y., Louis H., Benjamin I., Quaisie J., Duan Y., Tuly J.A., Cai M., Zhang H. (2024). Novel antioxidant peptides identified from coix seed by molecular docking, quantum chemical calculations and invitro study in HepG2 cells. Food Chem..

[69] Liu W., Ren J., Wu H., Zhang X., Han L., Gu R. (2023). Inhibitory effects and action mechanism of five antioxidant peptides derived from wheat gluten on cells oxidative stress injury. Food Biosci..

[70] Tao L., Gu F., Liu Y., Yang M., Wu X.-Z., Sheng J., Tian Y. (2022). Preparation of antioxidant peptides from Moringa oleifera leaves and their protection against oxidative damage in HepG2 cells. Front. Nutr..

[71] Fan H., Bhullar K.S., Wang Z., Wu J. (2022). Soybean-Derived Tripeptide Leu–Ser–Trp (LSW) Protects Human Vascular Endothelial Cells from TNFα-Induced Oxidative Stress and Inflammation via Modulating TNFα Receptors and SIRT1. Foods.

[72] Chen P., Huang P., Liang Y., Wang Q., Miao J. (2024). The antioxidant peptides from walnut protein hydrolysates and their protective activity against alcoholic injury. Food Funct..

[73] Murray C.J.L., Ikuta K.S., Sharara F., Swetschinski L., Robles Aguilar G., Gray A., Han C., Bisignano C., Rao P., Wool E. (2022). Global burden of bacterial antimicrobial resistance in 2019: A systematic analysis. Lancet.

[74] Bucataru C., Ciobanasu C. (2024). Antimicrobial peptides: Opportunities and challenges in overcoming resistance. Microbiol. Res..

[75] Li J., Hu S., Jian W., Xie C., Yang X. (2021). Plant antimicrobial peptides: Structures, functions, and applications. Bot. Stud..

[76] Karami Z., Muangnapoh C., Suthienkul O., Suriyarak S., Duangmal K. (2024). Exploring novel peptides in adzuki bean and mung bean hydrolysates with potent antibacterial activity. Int. J. Food Sci. Technol..

[77] Kong X., Song W., Hua Y., Li X., Chen Y., Zhang C., Chen Y. (2020). Insights into the antibacterial activity of cottonseed protein-derived peptide against Escherichia coli. Food Funct..

[78] Hu Y., Ling Y., Qin Z., Huang J., Jian L., Ren D.F. (2024). Isolation, identification, and synergistic mechanism of a novel antimicrobial peptide and phenolic compound from fermented walnut meal and their application in Rosa roxbughii Tratt spoilage fungus. Food Chem..

[79] Garzón A.G., Veras F.F., Brandelli A., Drago S.R. (2022). Purification, identification and in silico studies of antioxidant, antidiabetogenic and antibacterial peptides obtained from sorghum spent grain hydrolysate. LWT.

[80] Zhao Q., He L., Wang X., Ding X., Li L., Tian Y., Huang A. (2022). Characterization of a Novel Antimicrobial Peptide Isolated from *Moringa oleifera* Seed Protein Hydrolysates and Its Membrane Damaging Effects on *Staphylococcus aureus*. J. Agric. Food Chem..

[81] Sollazzo F., Di Nitto M., Rosito L., Torino F., Alvaro R., Lacarbonara F., Vellone E., Durante A. (2023). Caregivers’ contribution to self-care for patients treated with oral anticancer agents: A qualitative descriptive study. Eur. J. Oncol. Nurs..

[82] Norouzi P., Mirmohammadi M., Houshdar Tehrani M.H. (2022). Anticancer peptides mechanisms, simple and complex. Chem. Biol. Interact..

[83] Li M., Zhang Y., Xia S., Ding X. (2019). Finding and isolation of novel peptides with anti-proliferation ability of hepatocellular carcinoma cells from mung bean protein hydrolysates. J. Funct. Foods.

[84] Chen Z., Li W., Santhanam R.K., Wang C., Gao X., Chen Y., Wang C., Xu L., Chen H. (2019). Bioactive peptide with antioxidant and anticancer activities from black soybean [*Glycine max* (L.) Merr.] byproduct: Isolation, identification and molecular docking study. Eur. Food Res. Technol..

[85] Taghizadeh S.F., Azizi M., Asili J., Madarshahi F.S., Rakhshandeh H., Fujii Y. (2021). Therapeutic peptides of *Mucuna pruriens* L.: Anti-genotoxic molecules against human hepatocellular carcinoma and hepatitis C virus. Food Sci. Nutr..

[86] Gupta N., Bhagyawant S.S. (2021). Bioactive peptide of *Cicer arietinum* L. induces apoptosis in human endometrial cancer via DNA fragmentation and cell cycle arrest. 3 Biotech.

[87] Visuddho V., Halim P., Helen H., Muhar A.M., Iqhrammullah M., Mayulu N., Surya R., Tjandrawinata R.R., Ribeiro R.I., Tallei T.E. (2024). Modulation of Apoptotic, Cell Cycle, DNA Repair, and Senescence Pathways by Marine Algae Peptides in Cancer Therapy. Mar. Drugs.

[88] Wu B., Cheng H., Li X., Yang Q., Hao S., Wang C., Sun B. (2024). Identification and functional analysis of phycocyanin-derived bioactive peptides with non-small cell lung cancer cell inhibition. Algal Res..

[89] Du X., Xiao S., Luo Q., Liu X., Liu J. (2022). *Laminaria japonica* cyclic peptides exert anti-colorectal carcinoma effects through apoptosis induction in vitro and in vivo. J. Pept. Sci..

[90] Rasaratnam K., Nantasenamat C., Phaonakrop N., Roytrakul S., Tanyong D. (2021). A novel peptide isolated from garlic shows anticancer effect against leukemic cell lines via interaction with Bcl-2 family proteins. Chem. Biol. Drug Des..

[91] Wattayagorn V., Kongsema M., Tadakittisarn S., Chumnanpuen P. (2022). Riceberry rice bran protein hydrolyzed fractions induced apoptosis, senescence and G1/S cell cycle arrest in human colon cancer cell lines. Appl. Sci..

[92] Rout A., Duhan S., Umer M., Li M., Kalra D. (2024). Atherosclerotic cardiovascular disease risk prediction: Current state-of-the-art. Heart.

[93] Bregonzio C. (2022). Angiotensin-converting enzyme inhibitors stimulate cerebral arteriogenesis. Acta Physiol..

[94] Windarto S., Lee M.-C., Nursyam H., Hsu J.-L. (2022). First Report of Screening of Novel Angiotensin-I Converting Enzyme Inhibitory Peptides Derived from the Red Alga Acrochaetium sp. Mar. Biotechnol..

[95] Duan X., Dong Y., Zhang M., Li Z., Bu G., Chen F. (2023). Identification and molecular interactions of novel ACE inhibitory peptides from rapeseed protein. Food Chem..

[96] Zou Z., Wang M., Wang Z., Aluko R.E., He R. (2020). Antihypertensive and antioxidant activities of enzymatic wheat bran protein hydrolysates. J. Food Biochem..

[97] Li X., Peng C., Xiao S., Wang Q., Zhou A. (2024). Two Novel Angiotensin-Converting Enzyme (ACE) Inhibitory and ACE2 Upregulating Peptides from the Hydrolysate of Pumpkin (*Cucurbita moschata*) Seed Meal. J. Agric. Food Chem..

[98] Qoms M.S., Arulrajah B., Ibadullah W.Z.W., Ramli N.S., Shamsudin R., Chau D.-M., Saari N. (2024). Antihypertensive, Antidiabetic, and Antioxidant Properties of Novel Azolla pinnata Fern Protein Hydrolysates: Inhibition Mechanism, Stability, Profiling, and Molecular Docking. Food Bioprocess Technol..

[99] Bernier M.-È., Thibodeau J., Bazinet L. (2024). Enzymatic Hydrolysis of Water Lentil (Duckweed): An Emerging Source of Proteins for the Production of Antihypertensive Fractions. Foods.

[100] Zhang L., Pan D., Shao L., Zheng Y., Hao W., Kan Y., Cao J., Yu H., Liu J. (2023). Oil palm kernel globulin antihypertensive peptides: Isolation and characterization, ACE inhibition mechanisms, zinc-chelating activity, security and stability. Front. Pharmacol..

[101] Prangthip P., Panbangred W., Reamtong O. (2023). Potential antihypertensive activity of novel peptides from green basil leaves. BMC Complement. Med. Ther..

[102] Fan X., Ma X., Maimaitiyiming R., Aihaiti A., Yang J., Li X., Wang X., Pang G., Liu X., Qiu C. (2023). Study on the preparation process of quinoa anti-hypertensive peptide and its stability. Front. Nutr..

[103] Deng X., Peng D., Yao Y., Huang K., Wang J., Ma Z., Fu J., Xu Y. (2024). Optogenetic therapeutic strategies for diabetes mellitus. J. Diabetes.

[104] Chan-Zapata I., Sandoval-Castro C., Segura-Campos M.R. (2022). Proteins and peptides from vegetable food sources as therapeutic adjuvants for the type 2 diabetes mellitus. Crit. Rev. Food Sci. Nutr..

[105] Cai L., Wu S., Jia C., Cui C., Sun-Waterhouse D. (2023). Active peptides with hypoglycemic effect obtained from hemp (*Cannabis sativa* L) protein through identification, molecular docking, and virtual screening. Food Chem..

[106] Yuan X., Li P., Xiao Z.-W., Lou W.-Y. (2024). Preparation and identification of hypoglycemic bioactive peptide from *Amygdalus communis* L. by multienzyme hydrolysis. Process Biochem..

[107] Zhang Y., Wu F., He Z., Fang X., Liu X. (2023). Optimization and Molecular Mechanism of Novel α-Glucosidase Inhibitory Peptides Derived from Camellia Seed Cake through Enzymatic Hydrolysis. Foods.

[108] Xu F., Mejia E.G.d., Chen H., Rebecca K., Pan M., He R., Yao Y., Wang L., Ju X. (2020). Assessment of the DPP-IV inhibitory activity of a novel octapeptide derived from rapeseed using Caco-2 cell monolayers and molecular docking analysis. J. Food Biochem..

[109] Fidor A., Konkel R., Mazur-Marzec H. (2019). Bioactive Peptides Produced by Cyanobacteria of the Genus Nostoc: A Review. Mar. Drugs.

[110] Xu Y., Yang Y., Ma C.-m., Bian X., Liu X.-f., Wang Y., Chen F.-l., Wang B., Zhang G., Zhang N. (2023). Characterization of the structure, antioxidant activity and hypoglycemic activity of soy (*Glycine max* L.) protein hydrolysates. Food Res. Int..

[111] Sangeetha Vijayan P., Xavier J., Valappil M.P. (2024). A review of immune modulators and immunotherapy in infectious diseases. Mol. Cell. Biochem..

[112] Chalamaiah M., Yu W., Wu J. (2018). Immunomodulatory and anticancer protein hydrolysates (peptides) from food proteins: A review. Food Chem..

[113] Wen L., Jiang Y., Zhou X., Bi H., Yang B. (2021). Structure identification of soybean peptides and their immunomodulatory activity. Food Chem..

[114] Zhuang W., Lu X., Zheng Y., Song H., Zheng B. (2024). Immunomodulatory effects of lotus seed (*Nelumbo nucifera Gaertn*.) peptides on macrophages in mice. Food Biosci..

[115] Xu Z., Mao T.-M., Huang L., Yu Z.-C., Yin B., Chen M.-L., Cheng Y.-H. (2019). Purification and identification immunomodulatory peptide from rice protein hydrolysates. Food Agric. Immunol..

[116] Montserrat-de la Paz S., Villanueva-Lazo A., Millan F., Martin-Santiago V., Rivero-Pino F., Millan-Linares M.C. (2023). Production and identification of immunomodulatory peptides in intestine cells obtained from hemp industrial by-products. Food Res. Int..

[117] He P., Pan L., Wu H., Zhang L., Zhang Y., Zhang Y., Yang J., Lin Z., Zhang M. (2022). Isolation, Identification, and Immunomodulatory Mechanism of Peptides from Lepidium meyenii (Maca) Protein Hydrolysate. J. Agric. Food Chem..

[118] Liu P., Liao W., Qi X., Yu W., Wu J. (2020). Identification of immunomodulatory peptides from zein hydrolysates. Eur. Food Res. Technol..

[119] Liu H., Wang J., Liu Y., Bi H., Zhou X., Wen L., Yang B. (2024). Characterisation of functional pea protein hydrolysates and their immunomodulatory activity. Int. J. Food Sci. Technol..

[120] Segrist E., Cherry S. (2020). Using Diverse Model Systems to Define Intestinal Epithelial Defenses to Enteric Viral Infections. Cell Host Microbe.

[121] Zhou Y., Zhang D., Cheng H., Wu J., Liu J., Feng W., Peng C. (2024). Repairing gut barrier by traditional Chinese medicine: Roles of gut microbiota. Front. Cell. Infect. Microbiol..

[122] Wijesekara T., Abeyrathne E.D., Ahn D.U. (2024). Effect of Bioactive Peptides on Gut Microbiota and Their Relations to Human Health. Foods.

[123] Zhi T., Hong D., Zhang Z., Li S., Xia J., Wang C., Wu Y., Jia Y., Ma A. (2022). Anti-inflammatory and gut microbiota regulatory effects of walnut protein derived peptide LPF in vivo. Food Res. Int..

[124] Li L., Wang S., Zhang T., Lv B., Jin Y., Wang Y., Chen X., Li N., Han N., Wu Y. (2023). Walnut peptide alleviates obesity, inflammation and dyslipidemia in mice fed a high-fat diet by modulating the intestinal flora and metabolites. Front. Immunol..

[125] Zhang X., Jia L., He H., Yin H., Ming J., Hou T., Xiang J. (2022). Modulation of oxidative stress and gut microbiota by selenium-containing peptides from Cardamine enshiensis and structural-based characterization. Food Chem..

[126] Zhang T., Shi J., Liang F., Shi Y., Zhang R. (2022). Changes in biological activity and gut microbiota of digestion of rice glutelin during storage. J. Cereal Sci..

[127] Han C., Kong X., Xia X., Huang X., Mao Z., Han J., Shi F., Liang Y., Wang A., Zhang F. (2023). Effects of ginseng peptides on the hypoglycemic activity and gut microbiota of a type 2 diabetes mellitus mice model. J. Funct. Foods.

[128] Li W., Li H., Zhang Y., Zhang C., Zhang J., Liu X. (2021). Differences in the gut microbiota composition of rats fed with soybean protein and their derived peptides. J. Food Sci..

[129] Ren D., Wang P., Liu C., Wang J., Liu X., Liu J., Min W. (2018). Hazelnut protein-derived peptide LDAPGHR shows anti-inflammatory activity on LPS-induced RAW264.7 macrophage. J. Funct. Foods.

[130] Feng T., Huang Y., Tang Z., Wei D., Mo J. (2021). Anti-fatigue effects of pea (*Pisum sativum* L.) peptides prepared by compound protease. J. Food Sci. Technol..

[131] Wang S., Zheng L., Zhao T., Zhang Q., Liu Y., Sun B., Su G., Zhao M. (2020). Inhibitory Effects of Walnut (*Juglans regia*) Peptides on Neuroinflammation and Oxidative Stress in Lipopolysaccharide-Induced Cognitive Impairment Mice. J. Agric. Food Chem..

[132] Wang F., Yang G., Li Y., Tang Z., Du J., Song H., Xiong L., Wang L., Weng Z., Shen X. (2022). A peptide from wheat germ abolishes the senile osteoporosis by regulating OPG/RANKL/RANK/TRAF6 signaling pathway. Phytomedicine.

[133] Yuan X., Bao X., Liu X., Li X. (2022). Flaxseed-derived peptides ameliorate hepatic cholesterol metabolism in Sprague–Dawley rats fed a high-cholesterol and high-fat diet. J. Sci. Food Agric..

[134] Lu H., Fang L., Wang X., Wu D., Liu C., Liu X., Wang J., Gao Y., Min W. (2022). Structure-Activity Relationship of Pine Nut-Derived Peptides and Their Protective Effect on Nerve-Cell Mitochondria. Foods.

[135] Wen C., Zhang J., Zhang H., Duan Y., Ma H. (2021). Study on the structure–activity relationship of watermelon seed antioxidant peptides by using molecular simulations. Food Chem..

[136] Li W., Li H., Li J., Xu J., Fang L., Liu C., Wu D., Min W. (2023). Structure-activity relationship of walnut peptide in gastrointestinal digestion, absorption and antioxidant activity. LWT.

[137] Zhou Y., She X., Chen Z., Wei Y., Xiao Y., Zhou X. (2022). Tartary buckwheat (*Fagopyrum tataricum* (L.) Gaertn) protein-derived antioxidant peptides: Mechanisms of action and structure-activity relationship in Caco-2 cell models. Food Sci. Hum. Wellness.

[138] Zhu F., He S., Ni C., Wu Y., Wu H., Wen L. (2024). Study on the structure–activity relationship of rice immunopeptides based on molecular docking. Food Chem. X.

[139] Hsieh L.-S., Lu M.-S., Chiang W.-D. (2022). Identification and characterization of immunomodulatory peptides from pepsin–soy protein hydrolysates. Bioresour. Bioprocess..

[140] Velliquette R.A., Fast D.J., Maly E.R., Alashi A.M., Aluko R.E. (2020). Enzymatically derived sunflower protein hydrolysate and peptides inhibit NFκB and promote monocyte differentiation to a dendritic cell phenotype. Food Chem..

[141] Kang Y.-A., Kim Y.-J., Jin S.-K., Choi H.-J. (2023). Antioxidant, Collagenase Inhibitory, and Antibacterial Effects of Bioactive Peptides Derived from Enzymatic Hydrolysate of Ulva australis. Mar. Drugs.

[142] León Madrazo A., Fuentes Ortíz A.B., Morales Mendoza L.F., Segura Campos M.R. (2022). Antibacterial peptide fractions from chia seeds (*Salvia hispanica* L.) and their stability to food processing conditions. J. Food Sci. Technol..

[143] Xie J., Hong Z., Dai J., Li T., Bai Y., Zhang L., Hu X., Chen J., Sheng J., Tian Y. (2024). Isolation and identification of anti-colorectal cancer peptides from walnut proteins and associated in silico analysis. J. Funct. Foods.

[144] Taniya M., Reshma M., Shanimol P., Krishnan G., Priya S. (2020). Bioactive peptides from amaranth seed protein hydrolysates induced apoptosis and antimigratory effects in breast cancer cells. Food Biosci..

[145] Hu R., Xu J., Qi G., Wang W., Sun X.S., Li Y. (2022). Antioxidative hydrolysates from corn gluten meal may effectively reduce lipid oxidation and inhibit HepG2 cancer cell growth. J. Agric. Food Res..

[146] Phyo S.H., Ghamry M., Bao G., Zeng A., Zhao W. (2024). Potential inhibitory effect of highland barley protein hydrolysates on the formation of advanced glycation end-products (AGEs): A mechanism study. Int. J. Biol. Macromol..

[147] Cai L., Wu S., Jia C., Cui C. (2023). Hydrolysates of hemp (*Cannabis sativa* L.) seed meal: Characterization and their inhibitory effect on α-glucosidase activity and glucose transport in Caco-2 cells. Ind. Crops Prod..

[148] Chen H.-J., Dai F.-J., Chen C.-Y., Fan S.-L., Zheng J.-H., Chau C.-F., Lin Y.-S., Chen C.-S. (2023). Effects of molecular weight fraction on antioxidation capacity of rice protein hydrolysates. Sci. Rep..

[149] Yu X., Chen Y., Qi Z., Chen Q., Cao Y., Kong Q. (2023). Preparation and identification of a novel peptide with high antioxidant activity from corn gluten meal. Food Chem..

[150] López-Huertas E., Rubí-Villegas J., Sánchez-Moreno L., Nieto R. (2024). Olive Pomace Extract Contains Low Molecular Weight Peptides and Possesses ACE Inhibitory Activity. Int. J. Mol. Sci..

[151] Fang J., Lu J., Zhang Y., Wang J., Wang S., Fan H., Zhang J., Dai W., Gao J., Yu H. (2021). Structural properties, antioxidant and immune activities of low molecular weight peptides from soybean dregs (Okara). Food Chem. X.

[152] Dhaouafi J., Romdhani M., Deracinois B., Flahaut C., Nedjar N., Balti R. (2024). Fractionation and identification of bioactive peptides from red macroalgae protein hydrolysates: In silico analysis and in vitro bioactivities. Biocatal. Agric. Biotechnol..

[153] Wang H., Chi X., Zhang D. (2024). Potential Regulatory Gene Network Associated with the Ameliorative Effect of Oat Antibacterial Peptides on Rat Colitis. Foods.

[154] César A.P.C., Lopes F.E.S., Azevedo F.F.N., Pinto Y.O., Andrade C.R., Mesquita F.P., Silva G.O., Freitas C.D.T., Souza P.F.N. (2024). Antioxidant peptides from plants: A review. Phytochem. Rev..

[155] Abdelhameed S.A.M., Vandebroek L., de Azambuja F., Parac-Vogt T.N. (2020). Redox Activity of Ce(IV)-Substituted Polyoxometalates toward Amino Acids and Peptides. Inorg. Chem..

[156] Zhou N., Zhong Y., Liu H. (2024). Characterization and relationship analysis of antioxidant and anti-inflammatory peptides in pomelo fruitlet albumin. Food Chem..

[157] Wang X., Fu J., Bhullar K.S., Chen B., Liu H., Zhang Y., Wang C., Liu C., Su D., Ma X. (2024). Identification, in silico selection, and mechanistic investigation of antioxidant peptides from corn gluten meal hydrolysate. Food Chem..

[158] Wang L., Ma M., Yu Z., Du S.-k. (2021). Preparation and identification of antioxidant peptides from cottonseed proteins. Food Chem..

[159] Guo H., Hao Y., Richel A., Everaert N., Chen Y., Liu M., Yang X., Ren G. (2020). Antihypertensive effect of quinoa protein under simulated gastrointestinal digestion and peptide characterization. J. Sci. Food Agric..

[160] Roy S., Agarwal T., Das A., Halder T., Upadhyaya G., Chaubey B., Ray S. (2023). The C-terminal stretch of glycine-rich proline-rich protein (SbGPRP1) from Sorghum bicolor serves as an antimicrobial peptide by targeting the bacterial outer membrane protein. Plant Mol. Biol..

[161] LiWang A., Porter L.L., Wang L.-P. (2021). Fold-switching proteins. Biopolymers.

[162] Zhang S., Li Z.-m., Feng Y., Yu S., Li Z., Zhang D., Wang C. (2023). DPP-IV Inhibitory Peptides from Coix Seed Prolamins: Release, Identification, and Analysis of the Interaction between Key Residues and Enzyme Domains. J. Agric. Food Chem..

[163] Habinshuti I., Mu T.-H., Zhang M. (2020). Ultrasound microwave-assisted enzymatic production and characterisation of antioxidant peptides from sweet potato protein. Ultrason. Sonochem..

[164] Tian S., Meng F., Du K. (2024). Physicochemical properties and structure characteristics of different molecular weight peptides from ultrasonic assisted papain hydrolysate of wheat germ albumin. Ind. Crops Prod..

[165] Wen C., Zhang J., Zhou J., Duan Y., Zhang H., Ma H. (2018). Effects of slit divergent ultrasound and enzymatic treatment on the structure and antioxidant activity of arrowhead protein. Ultrason. Sonochem..

[166] Zhang X., Du H., Xu Z., Wang Y., Guo X., Xiao H., Li Y. (2023). A novel alcalase-hydrolyzed soybean meal hydrolysates prepared using by-product material: Structure, function property, sensory property, and biological activity. Food Biosci..

[167] Sun C., Shan Y., Tang X., Han D., Wu X., Wu H., Hosseininezhad M. (2021). Effects of enzymatic hydrolysis on physicochemical property and antioxidant activity of mulberry (*Morus atropurpurea* Roxb.) leaf protein. Food Sci. Nutr..

[168] Han J., Li D., Guo C., Wang J., Xue S. (2022). Construction and Reliability and Validity Test of Home Care Assessment Scale for Elderly Patients with Chronic Diseases Based on Intelligent Medical Care. Mob. Inf. Syst..

[169] Formanowicz D. (2024). Pathomechanisms of Disturbances Underlying Chronic Disorders. Biomedicines.

[170] Khan S.S., Greenland P. (2020). Comprehensive Cardiovascular Health Promotion for Successful Prevention of Cardiovascular Disease. JAMA.

[171] Pappachan J.M., Fernandez C.J., Chacko E.C. (2019). Diabesity and antidiabetic drugs. Mol. Asp. Med..

[172] Liu G., Yang L., Chen G., Xu F., Yang F., Yu H., Li L., Dong X., Han J., Cao C. (2021). A Review on Drug Delivery System for Tumor Therapy. Front. Pharmacol..

[173] Luvián-Morales J., Varela-Castillo F.O., Flores-Cisneros L., Cetina-Pérez L., Castro-Eguiluz D. (2022). Functional foods modulating inflammation and metabolism in chronic diseases: A systematic review. Crit. Rev. Food Sci. Nutr..

[174] Amato A. (2023). Natural Compounds and Healthy Foods: Useful Tools against Onset and Progression of Chronic Diseases. Nutrients.

[175] Mahgoub S., Alagawany M., Nader M., Omar S.M., Abd El-Hack M.E., Swelum A., Elnesr S.S., Khafaga A.F., Taha A.E., Farag M.R. (2023). Recent Development in Bioactive Peptides from Plant and Animal Products and Their Impact on the Human Health. Food Rev. Int..

[176] Chai T.-T., Ee K.-Y., Kumar Thirumal D., Manan Abd F., Wong F.-C. (2021). Plant Bioactive Peptides: Current Status and Prospects Towards Use on Human Health. Protein Pept. Lett..

[177] Stuermer E.K., Besser M., Terberger N., Bachmann H.S., Severing A.-L. (2019). Side Effects of Frequently Used Antihypertensive Drugs on Wound Healing in vitro. Ski. Pharmacol. Physiol..

[178] Wang Y., Li Y., Ruan S., Lu F., Tian W., Ma H. (2021). Antihypertensive effect of rapeseed peptides and their potential in improving the effectiveness of captopril. J. Sci. Food Agric..

[179] Xingfei L., Shunshun P., Wenji Z., Lingli S., Qiuhua L., Ruohong C., Shili S. (2020). Properties of ACE inhibitory peptide prepared from protein in green tea residue and evaluation of its anti-hypertensive activity. Process Biochem..

[180] Shu Y., Cao X.-Y., Chen J. (2019). Preparation and antagonistic effect of ACE inhibitory peptide from cashew. J. Sci. Food Agric..

[181] Blahova J., Martiniakova M., Babikova M., Kovacova V., Mondockova V., Omelka R. (2021). Pharmaceutical Drugs and Natural Therapeutic Products for the Treatment of Type 2 Diabetes Mellitus. Pharmaceuticals.

[182] Deng F., Liang Y., Lei Y., Xiong S., Rong J., Hu Y. (2023). Development and Identification of Novel α-Glucosidase Inhibitory Peptides from Mulberry Leaves. Foods.

[183] Liu W., Li H., Wen Y., Liu Y., Wang J., Sun B. (2021). Molecular Mechanism for the α-Glucosidase Inhibitory Effect of Wheat Germ Peptides. J. Agric. Food Chem..

[184] Wang X., Fan Y., Xu F., Xie J., Gao X., Li L., Tian Y., Sheng J. (2022). Characterization of the structure, stability, and activity of hypoglycemic peptides from Moringa oleifera seed protein hydrolysates. Food Funct..

[185] Marquez-Rios E., Del-Toro-Sanchez Lizette C. (2018). Antioxidant Peptides from Terrestrial and Aquatic Plants Against Cancer. Curr. Protein Pept. Sci..

[186] Chiangjong W., Chutipongtanate S., Hongeng S. (2020). Anticancer peptide: Physicochemical property, functional aspect and trend in clinical application (Review). Int. J. Oncol..

[187] Ma K., Wang Z., Ju X., Huang J., He R. (2023). Rapeseed peptide inhibits HepG2 cell proliferation by regulating the mitochondrial and P53 signaling pathways. J. Sci. Food Agric..

[188] Kim H., Kim H.-T., Jung S.-H., Han J.W., Jo S., Kim I.-G., Kim R.-K., Kahm Y.-J., Choi T.-I., Kim C.-H. (2023). A Novel Anticancer Peptide Derived from Bryopsis plumosa Regulates Proliferation and Invasion in Non-Small Cell Lung Cancer Cells. Mar. Drugs.

[189] Luna-Vital D., González de Mejía E. (2018). Peptides from legumes with antigastrointestinal cancer potential: Current evidence for their molecular mechanisms. Curr. Opin. Food Sci..

[190] Taghizadeh M.S., Niazi A., Moghadam A., Afsharifar A.R. (2021). Novel bioactive peptides of Achillea eriophora show anticancer and antioxidant activities. Bioorg. Chem..

